# Energy Deposition around Swift Carbon-Ion Tracks in Liquid Water

**DOI:** 10.3390/ijms23116121

**Published:** 2022-05-30

**Authors:** Pablo de Vera, Simone Taioli, Paolo E. Trevisanutto, Maurizio Dapor, Isabel Abril, Stefano Simonucci, Rafael Garcia-Molina

**Affiliations:** 1 Departamento de Física, Centro de Investigación en Óptica y Nanofísica, Universidad de Murcia, 30100 Murcia, Spain; 2 European Centre for Theoretical Studies in Nuclear Physics and Related Areas (ECT*), Bruno Kessler Foundation, 38123 Povo, Italy; taioli@ectstar.eu (S.T.); dapor@ectstar.eu (M.D.); 3 Trento Institute for Fundamental Physics and Applications (TIFPA-INFN), 38123 Trento, Italy; 4 Dipartimento di Ingegneria, Unità di Ricerca di Fisica non Lineare e Modelli Matematici, Università Campus Bio-Medico, Via Alvaro del Portillo 21, 00154 Roma, Italy; petrevi@gmail.com; 5 Departament de Física Aplicada, Universitat d’Alacant, 03690 San Vicente del Raspeig, Spain; ias@ua.es; 6 Division of Physics, School of Science and Technology, Università di Camerino and INFN Sezione di Perugia, 06123 Perugia, Italy; stefano.simonucci@unicam.it

**Keywords:** carbon ion beams, hadrontherapy, nanoscale biodamage, liquid water, Monte Carlo simulation, scattering cross sections in the condensed phase

## Abstract

Energetic carbon ions are promising projectiles used for cancer radiotherapy. A thorough knowledge of how the energy of these ions is deposited in biological media (mainly composed of liquid water) is required. This can be attained by means of detailed computer simulations, both macroscopically (relevant for appropriately delivering the dose) and at the nanoscale (important for determining the inflicted radiobiological damage). The energy lost per unit path length (i.e., the so-called stopping power) of carbon ions is here theoretically calculated within the dielectric formalism from the excitation spectrum of liquid water obtained from two complementary approaches (one relying on an optical-data model and the other exclusively on *ab initio* calculations). In addition, the energy carried at the nanometre scale by the generated secondary electrons around the ion’s path is simulated by means of a detailed Monte Carlo code. For this purpose, we use the ion and electron cross sections calculated by means of state-of-the art approaches suited to take into account the condensed-phase nature of the liquid water target. As a result of these simulations, the radial dose around the ion’s path is obtained, as well as the distributions of clustered events in nanometric volumes similar to the dimensions of DNA convolutions, contributing to the biological damage for carbon ions in a wide energy range, covering from the plateau to the maximum of the Bragg peak.

## 1. Introduction

Liquid water makes up around 75–80% of the mass of soft human tissues [1]. As a consequence, it is widely considered as an appropriate surrogate of living tissue in experimental and computational studies of radiation dosimetry [2]. Understanding precisely how different types of radiation (photons, electrons, ions) interact with and deposit their energy in this material in its condensed phase is of great relevance for further developing radiotherapies against cancer, as well as for radiation protection purposes on Earth or from cosmic radiation during manned space travel [3]. This is especially true for the advanced modality of radiotherapy using accelerated ion beams (such as protons or carbon ions) known as hadrontherapy. This cutting-edge technique is much more efficient than conventional radiotherapy using X-ray or electron beams [4], mainly due to physico-chemical interactions which take place at very different space, energy and time scales [5].

From a macroscopic point of view, ion beams, contrary to photons, do not suffer significant angular deflection and have a very well defined penetration range in matter. This gives place to a characteristic depth-dose curve (known as the Bragg peak) where a large fraction of their energy is deposited towards the end of their trajectories [4]. This Bragg peak is particularly sharp for carbon ions, which are nowadays considered the most promising projectiles for hadrontherapy [6,7]. Due to this feature, deep-seated tumours close to sensitive organs such as the brain, eye or spinal cord can be treated without inflicting much damage to the healthy surrounding areas. However, the theoretical prediction of the precise location of the Bragg peak for beams of different energies is very sensitive to the average energy loss per unit path length of ions in tissue (i.e., the stopping power) [8]. Despite their importance for treatment planning, the absolute values of the stopping power of liquid water for light ions (and particularly for carbon ions) are still under debate [9,10,11,12,13].

Microscopically, the energy-loss patterns of ion beams in tissue also give rise to their enhanced relative biological effectiveness (RBE), i.e., their ability to kill cells more effectively than photons or electrons do for the same amount of delivered dose [2,4,14,15,16]. The high RBE of ion beams, particularly of carbon ions, is related to the generation of large numbers of secondary species (low-energy secondary electrons and chemically reactive species) along the ion path, which give place to their microscopic track-structure [2,5]. Among these species, the secondary electrons produced by ion-impact ionisation are especially relevant. These are generated with low kinetic energies (typically <100 eV), so they present ranges of a few nanometres in liquid water. This creates concentrated patterns of energy deposition (sharp and intense nanometric radial doses) and reactive chemical species around the ion path, having the dimensions of the sensitive DNA strands carrying out the cell genetic information. Electrons directly, as well as, indirectly, the free radicals generated by them, can induce complex patterns of damage in the DNA strands, which, at high densities, are difficult to repair by the cell machinery, inducing the cell death. Secondary electrons with energies above the excitation threshold of liquid water (7 eV) can damage biomolecules by electronic excitation and ionisation, while even those below the threshold can induce damage by dissociative electron attachment (DEA) [17].

As the RBE of ions is so sensitive to the level of complex damage induced at the nanoscale, any attempt to model it (by means of Monte Carlo track-structure simulations [2,18] or analytical approaches [19,20]) requires an accurate knowledge of the underlying probabilities (or cross sections) for the different physical interactions between electrons and water (elastic and inelastic scattering, DEA, etc.). As an alternative to the computational modelling approaches (with which we deal in the present work), experimental nanodosimetry has been also developed in the last decades in order to estimate the complex damage induced by radiation at the scales of the DNA molecules [21,22,23].

Even though cross sections for water have been intensively studied experimentally and theoretically [24,25,26,27], most of the information gathered corresponds to the molecules in the gas phase. However, it is important to consider how the interaction of electrons with water is influenced by the condensed-phase nature of the real biological environment. Unfortunately, experimental work on this regard is extremely difficult, as it is challenging to disentangle individual scattering mechanisms from the unavoidable multiple scattering [28,29]. Most of the current nanodosimetry approaches are designed to work on gaseous detectors and, moreover, they are typically sensitive only to ionising collisions [21,22,23].

On this context, theoretical approaches become extremely useful to study individual interaction processes in the condensed phase. Concerning the study of electronic interactions (the main responsible for the energy loss of charged particles in matter), the dielectric formalism together with optical-data models (which exploit the excitation spectrum of the condensed-phase material, encoded in its dielectric properties) have been established as reference methodologies [2,30] (alternatively, other procedures have been developed to estimate the cross sections in condensed matter starting from atomic and molecular data, such as the IAM-SCAR methodology [31,32]). Particularly, the Mermin dielectric function [33], (used within the so-called Mermin Energy Loss Function-Generalised Oscillator Strengths (MELF-GOS) method [30,34,35]), has demonstrated to be a very reliable approach to reproduce the experimental excitation spectrum of liquid water [36], and to deliver stopping powers [37,38], ionisation cross sections for ion beams [39,40], and excitation and ionisation cross sections for electron beams [41,42], in good agreement with the known experimental data. More recently, *ab initio* approaches based on linear-response time-dependent density functional theory (LR-TDDFT) have shown to be able to give an extremely accurate representation of the excitation spectrum of liquid water [43]. Regarding the elastic collisions, it has also been shown that first principles methods (based on the solution of the Dirac equation [44,45,46]) can shed light on the electron scattering in condensed-phase water [43]. These inelastic and elastic cross sections are the necessary input to perform detailed Monte Carlo simulations of ion-impact production and transport of electrons in liquid water [43].

The purpose of the present work is to present these theoretical models to describe the inelastic and elastic scattering of electrons generated by swift carbon ions in liquid water, and to use these outcomes to provide detailed Monte Carlo simulations of the track-structure of carbon ions in a wide energy range (going from the low kinetic energies typical from the Bragg peak region in hadrontherapy up to very large energies characteristic of the Bragg curve plateau or of cosmic radiation). Monte Carlo simulations can yield very useful information on the patterns of energy deposition (radial doses) and clustering of damaging events in nanometric targets of the size of two DNA convolutions. This study presents new results, including the accurate simulation of radial doses and calculated stopping powers (in better agreement with recent experimental determinations around the stopping maximum than previous estimates). These findings, together with previously obtained results on clustering of damaging events on the nanometre scale, provide important insights on the energy deposition mechanisms of carbon ions in liquid water.

The calculation of the electronic excitation spectrum of liquid water is introduced in Section 2.1, where both the MELF-GOS and the LR-TDDFT approaches are explained and compared. In Section 2.2, the dielectric formalism approach for obtaining the electronic interaction cross sections for swift ion beams is presented, yielding the stopping power and other related energy-loss quantities of liquid water for carbon ions, based on the two previous approximations to the electronic excitation spectrum. The calculation of secondary electron energy and angular distributions for carbon ions in liquid water is developed in Section 2.3. The interaction cross sections for the secondary electrons are obtained in Section 2.4. The treatment of the elastic scattering with water molecules and water molecule clusters (in an effort to include condensed-phase effects) are reviewed in Section 2.4.1 and Section 2.4.2 respectively. Then the method to extend the dielectric formalism to obtain electronic excitation and ionisation cross sections for low-energy electrons in liquid water is detailed in Section 2.4.3, exploiting both the MELF-GOS and *ab initio* excitation spectra. The previous findings allow the detailed Monte Carlo simulation of the carbon-ion track-structure in liquid water, discussed in Section 2.5. Several quantities of interest in radiobiology are evaluated in Section 3, namely, the radial doses delivered around the ion path and the distributions of complex damaging events, including ionisation and electronic excitation, together with DEA. The summary and conclusions of the work are given in Section 4. Occasionally, atomic units (a.u.) will be used when needed.

## 2. Materials and Methods

### 2.1. Theoretical Models for the Electronic Excitation Spectrum of Liquid Water

The complex dielectric function $\epsilon \left(k,E\right)=\epsilon_{1}\left(k,E\right)+i\epsilon_{2}\left(k,E\right)$ (where $\epsilon_{1}$ and $\epsilon_{2}$ correspond, respectively, to its dispersive and absorptive parts) provides a connection between measurable dielectric properties of a target material and its electronic response to external charged particles [47,48], as a function of the momentum ℏk and the energy E=ℏω transferred to the target by an external electromagnetic perturbation. In the condensed phase, the electronic excitation spectrum of the target is contained in the energy loss function ELF=Im−1ϵ(k,E), which is the crucial quantity that determines the inelastic scattering cross section and the electronic energy loss of charged particles, as will be explained in Section 2.2. However, the ELF must be known over a broad range of energy and momentum transfers, i.e., the Bethe surface. Experimentally, the ELF can be determined by irradiating the target with photon or charged particle beams and analysing the outgoing particle energy spectra at different scattering angles, which correspond to different momentum-transfers. However, with this kind of experiments it is not possible to obtain the entire Bethe surface due to multiple-scattering effects at large momentum transfers [49]. As a consequence, it is crucial to count on with theoretical estimates that allow us to know the ELF with sufficient accuracy over the entire (k,E)-plane.

The traditional approach to obtain the Bethe surface relies on optical-data models, in which the optical ELF(k=0,E) is taken from experimental data and extended to k≠0 by appropriate models [30], as discussed in Section 2.1.1. However, current implementations of linear-response time-dependent density functional theory (LR-TDDFT) allow to directly calculate the ELF of liquid water from first principles for finite values of the momentum transfer (Section 2.1.2), without the need to use any particular set of experimental data.

#### 2.1.1. MELF-GOS Optical-Data Model

An optical data model successfully applied to describe the energy-loss quantities of charged particles in many condensed-phase materials [34,37,41,42,50,51,52,53,54,55] is the so-called MELF-GOS (Mermin Energy Loss Function-Generalised Oscillator Strengths) method [30,34,35,56,57]. This model properly describes the electronic excitation spectrum of a condensed-phase target, as it is the case of liquid water. In this methodology, the contributions to the ELF coming from the excitation of the loosely-bound outer-shell electrons and from the atomic-like inner-shell electrons are splitted as:

(1) Im−1ϵk,E=Im−1ϵk,Eouter+Im−1ϵk,Einner.

The justification for this separation lies on the fact that only the outer-shell electrons feel the characteristic screening effects of the condensed phase, while the excitation spectrum of the inner-shells is mostly insensitive to the target’s phase and can be treated as resulting from isolated atoms. Therefore the inner-shell electrons, that preserve their atomic character, are described by atomic generalised-oscillator-strengths (GOS) in the hydrogenic approach, for which analytical expressions are available. In general, for a compound target A${}_{\nu_{1}}$B${}_{\nu_{2}}$…, the inner-shell contribution to the ELF is given by [58]:

(2) Im−1ϵk,Einner=Im−1ϵk,EGOS=2π2ℏ2e2NE∑jνj∑nℓdfnℓj(k,E)dEΘ(E−Bnℓj),

where N is the molecular density of the target, $\nu_{j}$ is the stoichiometric contribution of the different *j* elements in the compound, $df_{n\ell }^{j}\left(k,E\right)/dE$ and $B_{n\ell }^{j}$ are, respectively, the GOS and the ionisation energy of the (n,ℓ) sub-shell of the *j*-element of the target. $\Theta (E-B_{n\ell }^{j})$ is a step function that becomes null when the transferred energy is not enough to ionise a target atom (i.e., $E<B_{n\ell }^{j}$). For liquid water, the K-shell electrons of oxygen are considered to contribute to the inner electron excitation, with an ionisation energy $B_{1s}^{O}=540$ eV [59].

The outer-shell electron excitations are described by a weighted sum of Mermin-type energy loss functions (MELF):

(3) Im−1ϵk,Eouter=Im−1ϵk,EMELF=∑jAj(ℏωj)2Im−1ϵMk,E;ωj,γjΘ(E−Eth,j),

where the coefficients $A_{j}$, $\omega_{j}$ and $\gamma_{j}$ account for, respectively, the intensity, position and width of the features of the experimental ELF. The step function $\Theta (E-E_{\mathrm{th},j})$ makes the ELF to vanish at transferred energies *E* smaller than some threshold energy $E_{\mathrm{th},j}$, which for liquid water corresponds to its excitation threshold energy ($E_{\mathrm{th}}=$ 7 eV), below which electronic excitations are not possible. The Mermin dielectric function $\epsilon_{M}$ is given by [33]:

(4) $$\epsilon_{M}\left(k,E\right)=1+\frac{\left(1+i\hbar \gamma /E\right)\;[\epsilon_{L}\left(k,E+i\hbar \gamma \right)-1]}{1+\left(i\hbar \gamma /E\right)\;\left[\epsilon_{L}\left(k,E+i\hbar \gamma \right)-1\right]/\left[\epsilon_{L}\left(k,0\right)-1\right]}\;,$$

and represents an improvement over the Lindhard dielectric function $\epsilon_{L}$ [48,60], The latter is based on the homogeneous electron gas model, together with the random-phase approximation (RPA), which assumes that each target electron interacts with the average field generated by all the other electrons. This assumption neglects dissipative processes and gives place to collective excitations with infinite lifetime [61]. The Mermin dielectric function includes the finite lifetime of collective excitations (or plasmon damping), as well as the effects of inter-band transitions, which made this model more realistic [2].

Since for long wavelengths (k=0) the experimental optical data are more accurate, in the MELF-GOS method the values of $A_{j}$, $\hbar \omega_{j}$ and $\hbar \gamma_{j}$ in Equation (3) are determined by fitting the outer-shell ELF contribution to the available experimental optical spectrum by means of the following relation:

Im−1ϵk=0,Eouter≃Im−1ϵk≃0,Eexp=

(5) ∑jAj(ℏωj)2Im−1ϵMk=0,E;ωj,γjΘ(E−Eth,j)=∑jAjEℏγj[(ℏωj)2−E2]2+[Eℏγj)2Θ(E−Eth,j).

Here, we have used the fact that for k=0 the Mermin-type ELF is identical to the Drude-type ELF, which is explicitly written on the most right hand side of Equation (5). The consistency of the fitting procedure is checked by fulfilling the Kramers–Kronig and *f*-sum rules [62]. The convenience of this method is that it includes in a realistic way the electronic excitation spectrum of liquid water (including collective and individual electronic excitations), as well as many body, chemical and physical state effects.

**Figure 1:**
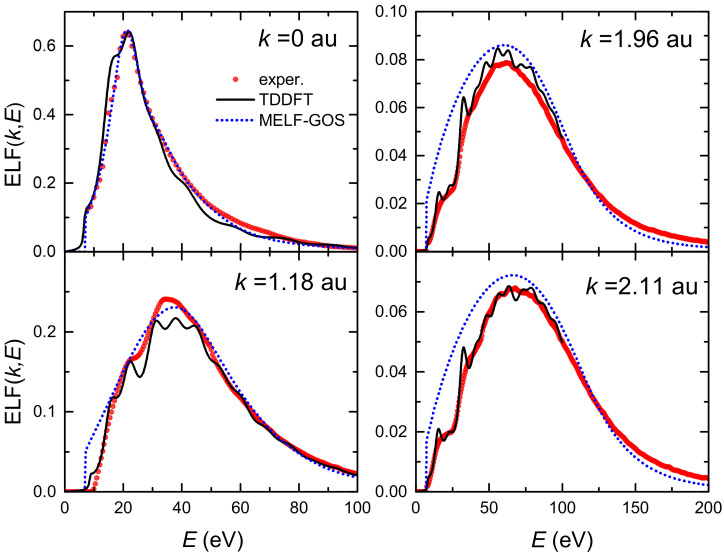
Energy loss function (ELF) of liquid water as a function of the energy transfer *E* at several momentum transfers ℏk from LR-TDDFT (black solid lines) and from MELF-GOS (blue dotted lines) approaches. Red circles correspond to experimental data [36,63,64].

The top left panel of Figure 1 depicts by red circles the experimental optical ELF (at k=0) measured from inelastic X-ray scattering spectroscopy [36,63,64], as well as the fitting made by means of the MELF-GOS method (dotted blue line). Subsequent panels show the calculated ELF for transferred momenta k= 1.18, 1.96 and 2.11 a.u., obtained from the analytical properties of the Mermin-type ELFs (without the need to introduce further assumptions about the dispersion relation) [30]. A broadening and reduction in the intensity of the ELF as the momentum transfer increases is observed, which agree with the theoretical expectation that individual excitations should gradually prevail over collective excitations for large momenta. The MELF-GOS results (dotted blue lines) agree fairly well with the experimental data (red circles) in a large range of energy transfers, which is one of the reasons why the MELF-GOS method is considered to lead to reliable energy-loss quantities for charged particles in liquid water.

From the MELF-GOS method it is also possible to calculate the mean excitation energy [62] of liquid water, which allows obtaining the ion or electron stopping powers at high projectile energies from the Bethe theory [65,66,67]. We obtain a value of I=79.4 eV [11], which is in agreement with recent recommendations [12,68].

#### 2.1.2. Linear-Response Time-Dependent Density Functional Theory

The polarisation function of the medium χ(k,E) can be determined by using linear-response time-dependent density functional theory (LR-TDDFT) by solving the equation [69]:

(6) $$\chi^{-1}\left(k,E\right)=\chi_{0}^{-1}\left(k,E\right)-v_{C}\left(k\right)-f_{\mathrm{xc}}\left(k,E\right)\;,$$

where $\chi_{0}^{-1}\left(k,E\right)$ is the non-interacting (or independent particle) polarisation calculated from the Kohn–Sham wavefunctions and band structures, $v_{C}\left(k\right)$ is the bare Coulomb interaction, and $f_{\mathrm{xc}}\left(k,E\right)$ is the TDDFT exchange and correlation kernel (usually Adiabatic Local Density Approximation (ALDA) but also the Adiabatic Perdew–Burke-Ernzerhof (APBE)). The microscopic dielectric matrix ϵ(k,E) is then related to the polarisation χ(k,E) by:

(7) $$\epsilon \left(k,E\right)=1-v_{C}\left(k\right)\chi \left(k,E\right)\;.$$

In the current calculations, we employ the APBE kernel [70] to obtain $f_{\mathrm{xc}}\left(r,t\right)$ which is related to the PBE exchange-correlation $v_{\mathrm{xc}}$ potential used in ground state density functional theory (DFT) calculations and the electronic density ρ(r,t) at the coordinates r and time *t* through:

(8) $$f_{\mathrm{xc}}\left(r,t\right)={\left\{\frac{d}{d\rho }v_{\mathrm{xc}}\left[\rho \right]\right\}}_{\rho =\rho (r,t)}\;.$$

Even though the Random Phase Approximation (RPA) ($f_{xc}=0$) provides a reasonable estimation of the macroscopic dielectric matrix, ALDA calculations have shown a general improvement in the agreement with the Inelastic X-Ray Scattering (IXS) experimental results, not only in finite-systems but also in crystalline systems [71,72,73,74,75]. This good TD-LDA (TD-DFT with ALDA kernel) behaviour in describing the IXS is commonly due to the less prominence of excitonic effects in the ELF in contrast to the absorption spectra (the ALDA and APBE omit the ultra-nonlocal term fundamental to represent them in the macroscopic limit, see Ref. [76]). Nevertheless, these approximations are not a general rule, and they must be checked for each system case by case (see, for instance, Ref. [77] where the excitonic effects are not negligible in ELF). The inclusion of self energy lifetimes in ALDA and APBE (TD-LDA+LT) has shown to improve the agree with the experiments in the high momentum transfer k regime [78,79]. In a periodic system, the inverse of the macroscopic dielectric function (which is the quantity to be compared to the experiment) is determined as

(9) $$\frac{1}{\epsilon_{M}\left(Q,E\right)}=\epsilon^{-1}{\left(k,E\right)}_{G,G},$$

where Q=k+G, with G being the reciprocal lattice vector of the target [80,81]. The energy loss function is then given by Im(ϵM−1). The off-diagonal elements of the dielectric matrix are responsible for the Local Fields Effects (LFEs) and become essential in inhomogeneous systems where localisation of atomic orbitals plays a significant role [75].

Calculations require previous generation and optimisation of a liquid water simulation box in the electronic ground state. Being an amorphous system, liquid water displays large degrees of randomness. To overcome the prohibitive generation of a statistical independent optimised water configurations ensemble, we assumed that a single snapshot of the liquid water configuration is enough to obtain its energy loss function ELF(k,E). This relies on previous photoabsorption spectra simulations of liquid water, where different molecular arrangements showed similar optical response [82].

A water supercell was generated by carrying out molecular dynamics (MD) simulations with several thousand molecules, using the empirical TIP3P force-field [83] implemented in the LAMMPS package [84]. The simulations ran for 100 ps, the first 10 ps being due to reach thermodynamic equilibrium at the temperature of 300 K. A cubic cell with side of 0.985 nm that can accommodate 32 water molecules to reproduce the experimental water density at room conditions (1 g/cm${}^{3}$) was then obtained. This cell size is a trade-off between reasonable computational effort of the many-body calculations and good agreement with experimental ELF data [36,63,64]. Finally, this cell was further relaxed imposing periodic boundary conditions below $10^{-3}$ Ry/Å for the interatomic forces via first-principles DFT calculations as implemented in the Quantum Espresso code suite [85], using PBE-GGA functionals [86] for both O and H to deal with the electron-electron Coulomb repulsion. To treat the ion-electron interaction we have used the Troullier–Martins (TM) norm-conserving pseudopotentials tabulated in the Quantum Espresso web page. Using the Γ point to sample the first Brillouin zone and a (kinetic) energy cut-off of 130 Ry, the self consistent DFT convergence is reached within the energy error of $10^{-5}$. It should be noted that, even though a recent study optimised liquid water samples by *ab initio* molecular dynamics [87], the current approach can be considered for all practical purposes equivalent (which will be seen from the results in the coming paragraphs), as our final classical molecular dynamics was also optimised by first principles prior to the ELF calculation.

First principles simulations of the ELF of liquid water for the optimised cell were carried out using the Lanczos chains algorithm (LCA) implemented in the turboEELS code [88]. LCA main advantage is that it allows to avoid the sum over the excited states. Calculations were performed in the energy range 0≤E≤100 eV for momentum transfers 0≤k≤2.5 a.u., with a resolution of 0.25 a.u. Due to the random orientation of water molecules, only the dependence on the wave vector module *k* was considered. The water ELF converged with a 4×4×4 Monkhorst-Pack mesh grid and 600 Lanczos iterations.

The LR-TDDFT results for the ELF of liquid water at various momentum transfers are shown by solid black lines in Figure 1, and are compared to the MELF-GOS method predictions (dotted blue lines) and the experimental data from X-ray scattering spectroscopy (red symbols) [36,63,64]. It can be seen that LR-TDDFT calculations give very good results at the optical limit (k=0), as well as an excellent description of the ELF evolution for finite momentum transfers. Particularly, the agreement with experiments at momentum values of 1.96 a.u. and 2.11 a.u. is remarkable, and better than the MELF-GOS predictions, which slightly overestimate the experimental ELF at large momenta. Noteworthy, current results are closer to the experimental data than similar LR-TDDFT calculations recently reported [87].

It should be noted, though, that despite the success of the LR-TDDFT calculations of the ELF of liquid water, *ab initio* determinations become prohibitive for energy transfers larger than 100 eV as well as for very large momentum transfers. Therefore, any further calculation of the ELF based on LR-TDDFT will require its extrapolation to E>100 eV and k>2.5 a.u. by means of the MELF-GOS methodology. The effect of using these two approaches to the ELF of liquid water on the energy-loss quantities for carbon ions and their secondary electrons will be analysed in the following sections.

### 2.2. Energy Loss of Swift Carbon Ions in Liquid Water

The dielectric formalism [47,66,89,90,91] represents the standard theoretical framework for studying the inelastic scattering of fast charged particles in condensed media, where the ELF of the material accounts, in an effective way, for the electronic excitation spectrum of the target in condensed phase. The model assumes that the perturbation produced on (and by) the moving charged particle is small and that it is possible to apply first order perturbation theory, i.e., that the particle both before and after scattering can be described by plane waves (the so-called first Born approximation, FBA). An important consequence of the dielectric formalism is that the differential inelastic scattering cross section can be factorised into a particle dependent (kinematic) factor and a material-dependent (dynamic) factor.

Let us consider a swift ion with net charge *q*, mass *M* and atomic number *Z* moving with kinetic energy *T* through a medium having a dielectric function ϵ(k,E). The electronic interactions are usually characterised by the energy *E* and momentum ℏk transferred in an inelastic collision between the incident ion and the target electrons, whose probability per unit path length $P_{q}^{i-e}\left(T,k,E\right)$ is given by [30] (the superscript “i−e” refers to the ion-electron interaction):

(10) Pqi−e(T,k,E)=d2Λqi−e(T,k,E)dEdk=e2πℏ2M[Z−ρq(k)]2T1kIm−1ϵ(k,E),

where *e* is the fundamental charge, $\rho_{q}\left(k\right)$ is the Fourier transform of the electronic charge density of the projectile of charge state *q* and Im[−1/ϵ(k,E)] is the energy loss function (ELF) of the material, Equation (1). In this work, the electronic charge density of the projectile is described by the statistical model proposed by Brandt and Kitagawa [92].

From a macroscopic point of view, Equation (10) corresponds to the inelastic doubly differential cross section (IDDCS), $d^{2}\Lambda_{q}^{i-e}\left(T,k,E\right)/dE\;dk$, from which one can obtain the statistical moments of the energy-loss distribution: the zeroth moment corresponds to the inverse inelastic mean free path (IIMFP), $\Lambda_{q}\left(T\right)$, the first moment to the stopping power, $S_{p,q}\left(T\right)$, and the second moment to the energy-loss straggling, $\Omega_{q}^{2}\left(T\right)$, i.e.,:

(11) $$M_{q}^{n}\left(T\right)=\int_{E_{-}}^{E_{+}}dE\;E^{n}\int_{k_{-}}^{k_{+}}dk\;P_{q}^{i-e}\left(T,k,E\right),$$

where the symbol $M_{q}^{n}\left(T\right)$ denotes the statistical moment of order *n* for the energy-loss distribution per unit path length of a projectile of charge state *q*. The IIMFP represents the average number of inelastic collisions experienced by the projectile per unit path length; the stopping power is the average energy lost per unit path length, and the energy-loss straggling is related to the width of the energy loss distribution.

The integration limits of Equation (11) are obtained by energy and momentum conservation in an inelastic collision. The lower limit for the energy transfer is $E_{-}=0$ if the target is a metal, or $E_{-}=E_{\mathrm{th}}$ (the excitation threshold energy) if it is a semiconductor or an insulator. For liquid water $E_{-}=E_{\mathrm{th}}$ = 7 eV. The upper limit in the energy transfer, assuming a collision with a free electron at rest, is $E_{+}=4\frac{m}{M}T$ [93] (where *m* is the electron mass), although the amount of energy transferred can be a little bit larger due to the recoil of the target. The limits for the momentum transfer are $\hbar k_{\pm }=\sqrt{2M}\left[\sqrt{T}\pm \sqrt{\left(T-E\right)}\right]$. For ion projectiles, as is the case for carbon ions, M≫m, and the following simplifications are possible: $\hbar k_{-}=E\sqrt{\frac{M}{2T}}$, $\hbar k_{+}\to \infty$ and $E_{+}\to \infty$.

**Figure 2:**
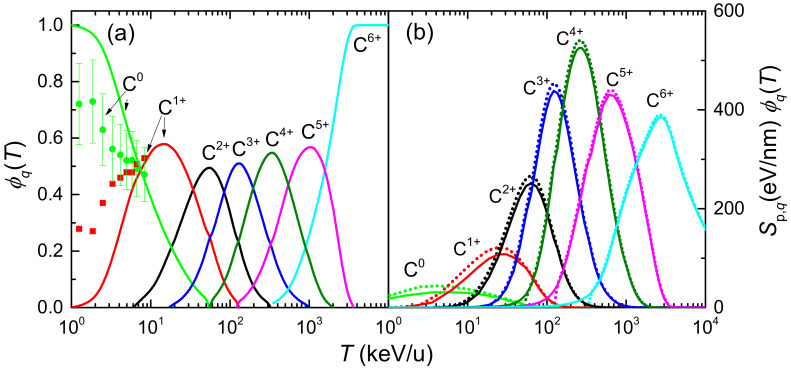
(**a**) Equilibrium charge state fractions, $\phi_{q}\left(T\right)$, of carbon ions in water, as a function of the incident energy *T*. Solid lines come from the parameterisation from Ref. [95] and Bragg’s rule, whereas symbols correspond to experimental data from [94]. (**b**) Stopping power multiplied by the equilibrium charge fraction, $S_{p,q}\left(T\right)\phi_{q}\left(T\right)$, for each charge state of carbon in liquid water, as a function of the incident energy. Solid (dotted) lines correspond to the LR-TDDFT (MELF-GOS) approach for the ELF.

On the other hand, it is necessary to consider that when a projectile travels through a condensed medium it can dynamically change its charge state due to electron capture and loss processes, which affect its energy loss. Therefore, after a few femtoseconds, when the projectile charge-state reaches a dynamical equilibrium, the total energy-loss quantities (Λ(T), $S_{p}\left(T\right)$, $\Omega^{2}\left(T\right)$) can be expressed as a weighted sum over their possible charge-states:

(12) $$M^{n}\left(T\right)=\sum_{q=0}^{Z}\phi_{q}\left(T\right)M_{q}^{n}\left(T\right)\;,$$

where $\phi_{q}\left(T\right)$ is the probability of finding the projectile in a given charge state *q* (the charge state fraction) at the energy *T*, which depends on the target nature, the projectile and its energy. Experimental data of equilibrium charge state fractions for carbon ions incident on water vapour are scarce, while those for liquid water do not exist. To our knowledge, only experimental data for C${}^{0}$ and C${}^{1+}$ in water molecule fragments have been obtained by time-of-flight mass spectrometry by Montenegro et al. [94], which are shown in Figure 2a by symbols. In our calculations, the equilibrium charge-state fractions as a function of the carbon projectile energy in water are obtained from a parameterisation to available experimental data from different targets and ions developed by Grande and Schiwietz [95] and where the Bragg’s additivity rule is employed for compound targets. The results of this approach are shown in Figure 2a by solid lines. Experimental [94] and parametric model [95] results agree around 10 keV/u; however, they deviate at lower collision energies. At energies larger than 3 MeV/u the parameterisation predicts fully stripped carbon ions. Note also the growing influence of small carbon charge fractions as the projectile energy decreases, which must be taken into account (according to Equation (12)) to calculate energy-loss quantities around the Bragg peak region energies. A classical trajectory Monte Carlo method was used by Liamsuwan and Nikjoo [96] to calculate the equilibrium charge fractions of carbon ions in water molecules, obtaining anomalously high values for C${}^{4+}$ fraction. Using these charge fractions in Equation (12), a stopping power was obtained with an unrealistic shoulder at energies lower than at the maximum $S_{p}$ [13]. It is clear that the charge state fractions strongly influence the calculated stopping power for ions such as carbon, which can be found in a large number of different charge states, particularly at energies around the maximum stopping power. Therefore, accurate experimental or theoretical determinations of the charge fractions of carbon ions in liquid water are extremely desirable.

Figure 2b represents the electronic stopping power of carbon ions impinging on liquid water for each charge state multiplied by their charge fraction, $S_{p,q}\left(T\right)\phi_{q}\left(T\right)$, as a function of the projectile energy *T*, as obtained from Equations (11) and (12). Solid (dotted) lines correspond to calculations using the ELF of liquid water derived from the LR-TDDFT (MELF-GOS) approach. Both values are quite similar, although small discrepancies appear around the maximum stopping power and are accentuated when the incident projectile energy decreases. This is a consequence of the differences between both calculated ELFs at the lower energy transfers *E*. For small charge fractions the stopping power calculated from the MELF-GOS method is systematically (moderately) larger than the results obtained from the LR-TDDFT model. We notice that at energies larger than 3 MeV/u only bare carbon ions contribute to the stopping. However, at intermediate energies around the maximum of the stopping power (∼200 keV/u), several intermediate charge states contribute. In view of these results, it can be deduced that the widely used assumption that the stopping power of heavy ions can be calculated from the proton stopping power through an effective charge $q_{\mathrm{eff}}$, such as $S_{p,\mathrm{heavy}\;\mathrm{ion}}=q_{\mathrm{eff}}^{2}S_{p,\mathrm{proton}}$, is not appropriate [97].

**Figure 3:**
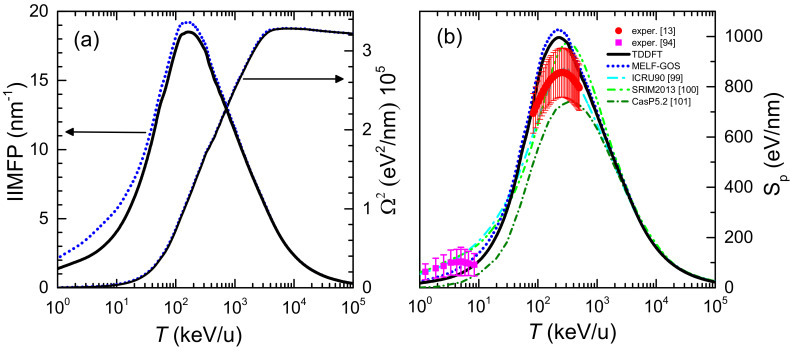
Energy-lossquantities of carbon ions in liquid water as a function of the incident projectile energy *T*. Black solid (blue dotted) lines correspond to our calculations when the liquid water ELF is described by the LR-TDDFT (MELF-GOS) approach. (**a**) Inverse inelastic mean free path (IIMFP) (left side axis) and energy-loss straggling $\Omega^{2}$ (right side axis). (**b**) Electronic stopping power. Available experimental data for liquid water are shown by red circles [13] and measurements for water molecules are depicted by magenta squares [94]. Other models and simulations are also shown (see the text for details).

The 0th and 2nd moments of the electronic energy-loss distribution of carbon ions in liquid water are depicted in Figure 3a as a function of the incident energy. All quantities have been weighted with the corresponding charge state fractions, Equation (12). Black solid (blue dotted) lines correspond to calculations based on the LR-TDDFT (MELF-GOS) approach to describe the ELF of liquid water. The left part of the axis in Figure 3a shows the IIMFP, which presents a maximum value at energies around 100–200 keV/u, corresponding to a mean free path of about 0.05 nm. At high projectile energies, the calculations obtained from the LR-TDDFT and MELF-GOS ELFs are rather similar, whereas at energies around and lower than the maximum IIMFP the results from MELF-GOS are slightly larger than the ones obtained from LR-TDDFT.

The electronic energy-loss straggling, $\Omega^{2}$, is presented in the right axis of Figure 3a. As the incident projectile energy increases, $\Omega^{2}$ grows, approaching a limiting value, known as the Bohr energy-loss straggling, $\Omega_{B}^{2}$ [35]. At high projectile energies and elemental targets of atomic number $Z_{t}$ it is possible to evaluate the Bohr straggling as $\Omega_{B}^{2}=4\pi e^{4}NZ^{2}Z_{t}$. Applying the additivity Bragg’s rule for compound targets, a value $\Omega_{B}^{2}=3.14\times 10^{5}$ eV${}^{2}$/nm is obtained, which is in good agreement with the calculated value. No differences are found between the energy-loss straggling obtained from the LR-TDDFT and the MELF-GOS ELFs in all the energy range.

The calculated electronic stopping power $S_{p}$ of carbon ions in liquid water is shown in Figure 3b for the LR-TDDFT (black solid line) and the MELF-GOS (blue dotted line) methodologies to describe the ELF of liquid water. Both approaches provide similar values for energies larger than ∼1 MeV/u. At lower energies, the MELF-GOS method systematically gives larger stopping power values than those obtained by the LR-TDDFT ELF; the largest discrepancy is ∼3% and occurs around the maximum stopping power at carbon energies ∼220 keV/u. Recently, a sophisticated experiment to measure the stopping power of carbon ions in liquid water (whose results are depicted by red circles in Figure 3b) has been performed [13] for energies in the range 1–6 MeV (around the maximum stopping power) using the inverted Doppler shift attenuation method with an improved experimental setup than in preliminary measurements [98]. These are the only experimental stopping power data available for carbon ions in liquid water around the maximum. Our theoretical stopping power calculations (see Figure 3b) present their maximum value at the same energies as the experiments [13], although with higher values, but close to the experimental error bars. The stopping power obtained with the LR-TDDFT model is closer to the experimental data, being only 5% higher than the upper limit of the experimental error bars. It is worth to recall at this point, in any case, the comment made in Ref. [99] regarding previous measurements by Baek et al. for carbon in graphite using the same inverted Doppler shift attenuation method, in which the normalisation method used may underestimate the absolute values by ∼15%. Although it does not seem that the same issue applies for the most recent determinations [13], such an increase applied to the experimental data would make it to almost perfectly match with our calculations based on both the MELF-GOS and the LR-TDDFT ELF, in shape as well as in absolute value. At low carbon energies, the stopping power for water molecule fragments has been measured by Montenegro et al. [94] (magenta square symbols in Figure 3b, which agree within the experimental uncertainties with the presented calculations. Although at these very low ion energies nuclear stopping power (not included in current calculations) may be important, an estimate using the semiempirical code SRIM2013 [100] shows that, for energies around 10 keV/u, nuclear stopping only contributes ∼10–15% to the total energy loss. Moreover, Montenegro et al. also measured electronic energy-loss, so their experimental data can be directly compared to our results. It should be noted that, for the integral energy-loss quantities, only small differences in the calculations are observed using the MELF-GOS or LR-TDDFT approaches to the ELF.

Due to the enormous importance of the stopping power in several areas of physics and materials science, several models or semiempirical approaches to predict it for a variety of ions, targets and energies have been developed. Figure 3b shows the recommended data by ICRU [99] by a cyan dash-dotted line, where the agreement with the available experimental data [13,94] is very good, as the ICRU compilation mainly relies on available experimental data. The semiempirical code SRIM2013 [100] is depicted by a green dash double-dotted line, which for compound targets is based on the Bragg’s additivity rule and where an extrapolation of the experimental stopping power for H, He and Li ions was used. This widely used code predicts stopping power values with the maximum shifted towards larger energies and (particularly) with higher values than reported in the experiments [13]. The results of the theoretical model CasP v.5.2 [101] are shown by a dark green dotted line. CasP employs a non-perturbative unitary convolution approximation (UCA) method to calculate the impact-parameter dependent energy loss for each target-subshell and for each projectile charge-state separately. In this calculation for carbon in water, CasP uses a value of *I* = 78 eV [68] for the mean excitation energy of liquid water. The stopping power calculated by the CasP code is lower than the other codes and the experimental data. All the calculated and semiempirical stopping powers practically merge at energies larger than 3 MeV/u.

### 2.3. Angular and Energy cross Sections of Electrons Generated by Energetic Carbon Ions

It is not sufficient to count with an accurate knowledge of the integral energy-loss quantities (such as the stopping power or the total number of emitted electrons, i.e., the total ionisation cross sections) to evaluate the biodamage produced in the target. The angular and energy distributions of the secondary electrons are also crucial to understand how the electron cascade transports the energy lost by the projectile around its path. Based on the dielectric response formalism (as explained in the previous section), a model has been developed [39,40] able to calculate, in a relatively simple way and with reasonable accuracy, the energy (i.e., the ionisation singly differential cross sections, ionis-SDCS) [39] and the angular distributions (i.e., the ionisation doubly differential cross sections, ionis-DDCS) of secondary electrons generated by the incidence of energetic ions in condensed targets [40]. The advantage of this model lies on its applicability to a wide range of energies and projectile-target combinations, especially in the condensed phase, and on its simplicity, which makes it easy to be implemented in radiobiological models, with a reasonable computing time.

Starting from Equation (10) and using the relation between the macroscopic Λ=Nσ and the microscopic σ cross sections, where N is the molecular density of the material, the total ionis-DDCS of an ion with energy *T* can be expressed as:

(13) d2σq,ionisi−e(T,k,W)dWdk=e2πℏ2NM[Z−ρq(k)]2T1k∑αIm−1ϵ(k,Bα+W)α,

where the sum goes over the different target electronic (both outer- and inner-) shells, i.e., α=outer/inner. The transferred energy in an ionising collision is expressed as $E=B_{\alpha }+W$, where *W* is the kinetic energy of the secondary electron and $B_{\alpha }$ is the binding energy of the α electronic shell. Im−1ϵ(k,Bα+W)α refers to the outer- or inner-shell contributions to the ELF, as defined in Equation (1). For liquid water we take the oxygen K-shell as an inner-shell with binding energy $B_{1s}^{O}$ = 540 eV [59]. For the outer-shells of liquid water (and, in general, for organic materials), an approximated mean binding energy was introduced, because the outer-shell (i.e., valence) ELF only presents a single clear excitation, due to the closeness among the binding energies of the different outer-shells [39]. For liquid water, the mean binding energy is *B* = 13.7 eV [42].

The energy spectrum of the emitted secondary electrons generated by an incident ion, $d\sigma_{q,\mathrm{ionis}}^{i-e}\left(T,W\right)/dW$, or the ionisation singly differential cross section (ionis-SDCS), is simply obtained by integrating Equation (13) over momentum transfers:

(14) $$\frac{d\sigma_{q,\mathrm{ionis}}^{i-e}\left(T,W\right)}{dW}=\int dk\frac{d^{2}\sigma_{\mathrm{ionis}}^{i-e}\left(T,k,W\right)}{dW\;dk}\;.$$

**Figure 4:**
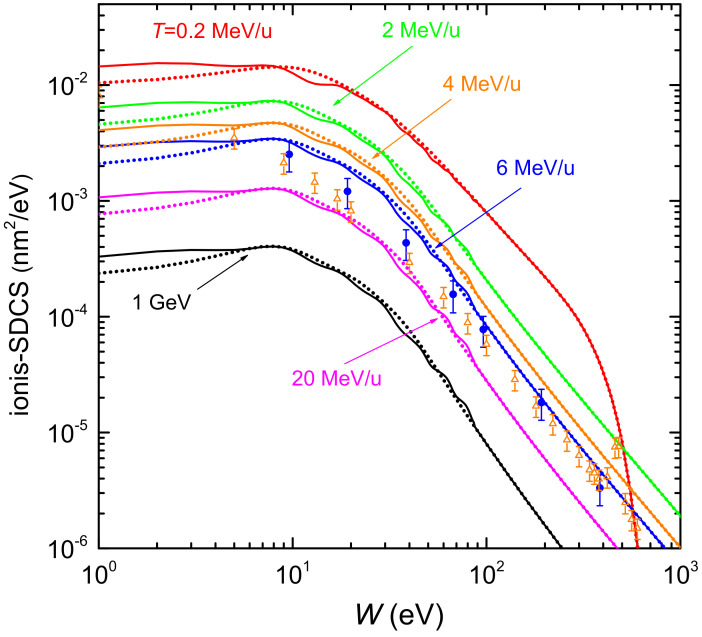
Energy distributions (ionis-SDCS) of emitted electrons as a function of the ejected kinetic energy *W*, for several carbon incident energies *T*. Solid (dotted) lines correspond to our calculations for liquid water using the LR-TDDFT (MELF-GOS) approach. Symbols are experimental data for water vapour coming from Ref. [26] (6 MeV/u carbon ions, full circles) and from Ref. [102] (4 MeV/u carbon ions, open triangles).

Figure 4 shows the distributions of energy *W* of the secondary electrons (ionis-SDCS) generated by carbon ions with kinetic energies from 0.2 MeV/u up to 1 GeV in liquid water. Solid (dotted) lines are calculations based on the LR-TDDFT (MELF-GOS) approach to evaluate the ELF of liquid water. At high energies *W* of the emitted electrons, both models give similar results and only at energies *W* less than 10 eV appreciable (but not significant) differences appear. The maximum value of the ionis-SDCS is for T=0.2 MeV/u, which corresponds to the energy at which the maximum stopping power occurs. It is interesting to remark that when the energy of the incoming carbon ion increases, the value of ionis-SDCS diminishes very quickly. For instance, when the energy *T* increases from 0.2 MeV/u to 20 MeV/u, the (maximum) value of ionis-SDCS decreases in almost a factor 14. It is also worth mentioning that the ionis-SDCS presents, for a given ion energy, an *W*-value at which the cross section drastically drops to zero. This corresponds to the kinematic limit, i.e., the maximum energy that an ion can transfer to an electron [93], which grows with the ion energy. For the case *T* = 0.2 MeV/u, this limit is seen in the figure at W∼600 eV. Circles in the figure correspond to experimental data in water vapour for T=6 MeV/u carbon ions [26], while triangles are for T=4 MeV/u [102]. The agreement between our results and the experimental data for 6 MeV/u is reasonable, especially considering the phase difference of the targets analysed. However, the experimental data for 4 MeV/u seem too low compared to our calculations. As it will be discussed in the following, there might be some scaling issue with the experimental data from Ref. [102], as a 4 MeV/u-ion should have a larger ionis-SDCS than a 6 MeV/u one.

The ionis-DDCS, in terms of the scattering angle $\theta_{1}$ of the incident ion, can be calculated taking into account the relationship between the momentum transfer ℏk in an inelastic collision and this angle:

(15) $$\hbar k=\sqrt{2M\left[2T-\left(B_{\alpha }+W\right)-2\sqrt{T(T-\left(B_{\alpha }+W\right))}\cos\theta_{1}\right]}\;.$$

However, our objective is to obtain the ionis-DDCS as a function of the energy *W* and the angle $\theta_{2}$ of the electron emitted in the inelastic collision. For this purpose, it was assumed [40] that both angles are proportional, $\theta_{1}=C\cdot \theta_{2}$, which means that the maximum position in ionis-DDCS($\theta_{2}$) is correlated with the maximum position in ionis-DDCS($\theta_{1}$), and that the shapes and widths of these distributions are proportional. To calculate *C* we take into account that the ionis-DDCS($\theta_{2}$) is dominated by the binary encounter peak, i.e., a well-defined maximum at $\theta_{2}^{\mathrm{BE}}=\arccos\sqrt{MW/(4mT)}$, where the collision can be regarded as a binary collision between free particles. On the other hand, the ionis-DDCS($\theta_{1}$) is also dominated by a maximum at $\theta_{1}^{\max}$. In principle, it is reasonable to assume that this maximum value corresponds to the binary encounter collision, so the proportionality constant for a projectile with mass *M* and kinetic energy *T* that ejects an electron with energy *W* is $C\left(M,T,W\right)=\theta_{1}^{\max}/\theta_{2}^{\mathrm{BE}}$.

If we express the ejection angle $\theta_{2}$ of the emitted electron as a function of the solid angle, $d\Omega_{2}=2\pi \sin\theta_{2}d\theta_{2}$, the ionis-DDCS(*W*, $\theta_{2}$) to eject electrons in the angle $\theta_{2}$, per unit energy *W* and solid angle $\Omega_{2}$ is [40]:

(16) d2σq,ionisi−edWdΩ2=Ce22π2ℏ2Nsinθ2M[Z−ρq(k)]2T∑αIm−1ϵ(k,Bα+W)α×T(T−(Bα+W))sinCθ22T−(Bα+W)−2T(T−(Bα+W))cosCθ2.

This equation, based on the first Born approximation (i.e., no interaction considered between the ejected electron and the scattered and residual ions), can be improved by taking into account two center effects (i.e., the ejected electron being attracted by the projectile after the collision) if we multiply it by the semiempirical Salin’s factor $F_{S}$, given by [93,103,104]:

(17) $$F_{S}=\frac{u}{1-e^{-u}}\;,$$

with:

(18) $$u=2\pi {B_{\alpha }}^{1/2}\left[{\left(\frac{T}{M}+W-2{\left(\frac{TW}{M}\right)}^{1/2}\cos\theta_{2}\right)}^{-1/2}-{\left(\frac{T}{M}\right)}^{-1/2}\right]\;.$$

Thus the number of electrons ejected in the forward direction (electron capture to the continuum) attracted by the field of the projectile after the collision is corrected, showing an improvement with the experimental data for small angles for H and He projectiles in several targets [40].

**Figure 5:**
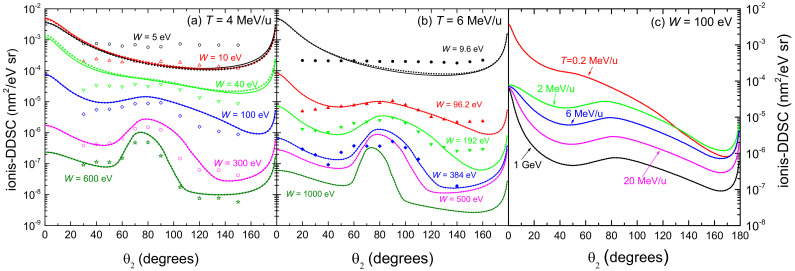
Angular distributions (ionis-DDCS) of emitted electrons by the impact of (**a**) 4 MeV/u and (**b**) 6 MeV/u carbon ions, as a function of the emitted angle $\theta_{2}$ at different values of the emission energy *W*. Solid (dotted) lines correspond to our calculations for liquid water using the LR-TDDFT (MELF-GOS) approach, while symbols are experimental data for 4 MeV/u (empty symbols [102]) and 6 MeV/u (full symbols [26]) carbon ions in water vapour. (**c**) Angular distribution (ionis-DDCS) of 100 eV electrons generated by the incidence of carbon ions at several energies *T* in liquid water.

Figure 5 represents the angular distribution (ionis-DDCS) of electrons ejected by the incidence of (a) 4 MeV/u and (b) 6 MeV/u C${}^{6+}$ ions in water, as a function of the emission angle $\theta_{2}$ at several energies *W* of the emitted electrons. The results obtained from the LR-TDDFT (solid lines) and from the MELF-GOS approaches (dotted lines) for liquid water are very similar in most of the cases, showing small differences only at very low electron energies. As the energy *W* of the emitted electrons increases, a maximum appears in the angular distribution, which corresponds to the binary encounter peak. Comparison with experimental data for 6 MeV/u carbon ions in water vapour [26] shows a good agreement except for very large angles and very low emitted energies. These discrepancies may be attributed to the phase difference between the experiments (gas) and the calculations (liquid). Regarding the experimental data fo 4 MeV/u carbon ions [102], the agreement is also good in terms of the shapes of the ionis-DDCS curves, although the experimental absolute values are systematically lower than our calculations for most of the emission energies *W* (except for the lower ones). As commented earlier, this might point out to a normalisation issue with this particular set of data [102]. Actually, similar conclusions were drawn in Ref. [105] when comparing their classical trajectory Monte Carlo calculations to these and other sets of ionis-SDCS and ionis-DDCS data for different projectiles.

Figure 5c shows the angular distribution (ionis-DDCS) of ejected electrons with energy W= 100 eV in liquid water, due to the impact of carbon ions having energies between 0.2 MeV/u and 1 GeV. The calculations have been convoluted with the energy-dependent equilibrium charge state fraction of the projectile (see Figure 2a), which show that projectiles with energies larger than 3–4 MeV/u travel through water as bare C${}^{6+}$ ions. Results obtained from the LR-TDDFT (solid lines) and from the MELF-GOS (dotted lines) approaches to the liquid water ELF are practically identical. At carbon energies larger than 2 MeV/u, the relative shapes of the angular distributions are similar, but for T= 0.2 MeV/u, the behaviour of the ionis-DDCS is more forward-peaked, probably due to the contributions in this case from the charge states from C${}^{2+}$ up to C${}^{5+}$.

### 2.4. Cross Sections for Electrons in Liquid Water

A secondary electron will suffer elastic and inelastic collisions with the water molecules until thermalising its energy and eventually becoming solvated or attached to the molecules in the medium. The elastic collisions will change the direction of motion of the electron, while the inelastic events will slow it down until stopping. In addition, as a result of the inelastic collisions, the energy lost by the electron may be locally deposited in the medium by electronic excitations, or it may lead to the ejection of another electron by ionisation, transporting the energy further away. These inelastic events can also fragment water molecules to produce chemically reactive species.

Elastic scattering of electrons by atomic cores can be dealt with using two different levels of theory: the relativistic Mott theory [106] with a potential taken as best fit of data from Hartree–Fock (HF) simulations, typically in a central field (Section 2.4.1), or the direct self-consistent solution of the Dirac equation, which can be extended to multi-centered potentials [46], such as in the case of liquid systems (Section 2.4.2).

The inelastic collisions lead to electronic excitations and ionisations, whose cross sections for electrons in liquid water will be obtained in Section 2.4.3 within the framework of the dielectric response theory, which will be also used to estimate the electron-induced probabilities for water molecule fragmentation.

#### 2.4.1. Elastic cross Section of an Electron with a Water Molecule Obtained by the Mott Theory

This approach gives access to the elastic differential scattering cross-section in the solid angle dΩ of an electron with an atomic core (here referred as “e−c” interaction), which in the case of scattering from a central potential can be written using relativistic quantum mechanics as [106,107,108,109,110]:

(19) $$\frac{d\sigma_{\mathrm{el}}^{e-c}}{d\Omega }\;={\;[|f\left(\theta \right)|}^{2}+{\left|g\left(\theta \right)\right|}^{2}]\left[1+S\left(\theta \right)P\cdot \hat{n}\right]\;,$$

where the subindex “el” refers to the elastic electron scattering. The functions f(θ) and g(θ) are the direct and spin-flip scattering amplitudes, respectively, and θ represents the scattering angle, S(θ) is the Sherman function and

(20) $$\hat{n}=\frac{k_{i}\times k_{f}}{|k_{i}\times k_{f}|}$$

is the versor orthogonal to both the initial ($\hbar k_{i}$) and final ($\hbar k_{f}$) momenta of the electrons, respectively. P=0(1) means that the beam emerges not (fully) polarised.

However, in our case being the target a water molecule, for which the central symmetry is broken, the previous derivation must be generalized to deal with the electron-molecule scattering. In the molecular case, one has:

(21) $$\frac{d\sigma_{\mathrm{el}}^{e-c}}{d\Omega }\;=\;\sum_{m,n}\;\exp\left(ik\cdot r_{mn}\right)\;\left[f_{m}\left(\theta \right)f_{n}^{*}\left(\theta \right)\;+\;g_{m}\left(\theta \right)g_{n}^{*}\left(\theta \right)\right]\;,$$

where ℏk is the momentum transfer, $r_{mn}\;=\;r_{m}\;-\;r_{n}$, with $r_{m}\left(r_{n}\right)$ being the position vector of the mth(nth)–atom in the molecule, and $f_{m}\left(\theta \right),g_{m}\left(\theta \right)$ are the direct and spin-flip scattering amplitudes of the mth–atom. As water molecules in liquid phase are randomly oriented, one can average over all the orientations. By performing this average, Equation (21) reads [108]:

(22) $$\frac{d\sigma_{\mathrm{el}}^{e-c}}{d\Omega }\;=\;\sum_{m,n}\;\frac{\sin kr_{mn}}{kr_{mn}}\;\left[f_{m}\left(\theta \right)f_{n}^{*}\left(\theta \right)\;+\;g_{m}\left(\theta \right)g_{n}^{*}\left(\theta \right)\right]\;.$$

Writing explicitly this expression for the water molecule, the elastic differential scattering cross-section (EDCS) of electrons impinging on randomly oriented water molecules is:

(23) $$\begin{array}{ccc} & & {\left(\frac{d\sigma_{\mathrm{el}}^{e-c}}{d\Omega }\right)}_{H_{2}O}\;=\;2\;{\left(\frac{d\sigma_{\mathrm{el}}^{e-c}}{d\Omega }\right)}_{H}\;+\;{\left(\frac{d\sigma_{\mathrm{el}}^{e-c}}{d\Omega }\right)}_{O}\;\\ & & +\;2\;\frac{\sin kr_{\mathrm{OH}}}{kr_{\mathrm{OH}}}\left[f_{H}\left(\theta \right)f_{O}^{*}\left(\theta \right)+f_{O}\left(\theta \right)f_{H}^{*}\left(\theta \right)\;+\;g_{H}\left(\theta \right)g_{O}^{*}\left(\theta \right)\;+\;g_{O}\left(\theta \right)g_{H}^{*}\left(\theta \right)\right]\;\\ & & +2\;\frac{\sin kr_{\mathrm{HH}}}{kr_{\mathrm{HH}}}\left[\right|f_{H}{\left(\theta \right)|}^{2}\;+\;|g_{H}\left(\theta \right){|}^{2}]\;, \end{array}$$

where $r_{\mathrm{OH}}\;$= 0.09572 nm and $r_{\mathrm{HH}}\;$= 0.1514 nm are the equilibrium bond lengths of the water molecule, $f_{O/H}\left(\theta \right)$, $g_{O/H}\left(\theta \right)$ are the direct and spin-flip scattering amplitudes of O and H. The first and second terms describe the independent atomic contribution to the EDCS, while the third and fourth terms include the interference between elastically scattered electron waves emerging from the atomic constituents of water.

The many-body electrostatic atomic potential in the Dirac equation was modelled by a screened Coulomb potential. The latter is obtained by multiplying a bare Coulomb potential by a function expressed as a superposition of Yukawa functions, whose parameters were set according to a best fit of data from Hartree–Fock simulations [107]. Exchange effects were described by using the Furness and McCarthy formula [111].

#### 2.4.2. Elastic cross Section of an Electron with Liquid Water Molecules Obtained by the First Principles Approach

The Dirac Hamiltonian of many electron systems with mass *m*, interacting via a Coulomb potential can be written in Hartree–Fock (HF) approximation [112]:

(24) $$\left(\begin{array}{cc} mc^{2}+V_{H}+V_{\mathrm{exc}}-E & -c\Sigma \cdot i\nabla \\ -c\Sigma \cdot i\nabla & -mc^{2}-V_{H}-V_{\mathrm{exc}}-E \end{array}\right)\left(\begin{array}{c} \psi_{L}\\ \psi_{S} \end{array}\right)=0,$$

where $\psi_{L}$ and $\psi_{S}$ are, respectively, the large and small components of the Dirac spinor, and $V_{H},V_{\mathrm{exc}}$ are the Hartree and non-local exchange terms. E is the energy, while Σ corresponds to the vector of the Pauli matrices. The numerical solution of the Dirac Hamiltonian, Equation (24), was found by defining a projector:

(25) $$\pi =\sum_{j}|g_{j}\rangle \langle g_{j}|\;,$$

onto a finite functional space G of $L^{2}$-functions $g_{j}$s. In our numerical model the functions $g_{j}$s are Gaussians. This approach is particularly suitable when dealing with molecular systems without spherical symmetry, such as the case of a liquid water cluster. The Dirac equation can be projected in this functional space as follows:

(26) $$\left(H_{0}-E+\pi V\pi \right)\psi =0\;,$$

where $H_{0}$ is the unperturbed hamiltonian (the kinetic energy in this case). This equation can be transformed into a Lippmann–Schwinger type of relation:

(27) $$\pi \psi =\pi \frac{1}{E-H_{0}}\pi V\pi \psi \;.$$

We notice that only the Coulomb potential in the Hamiltonian (26) is projected into the Hilbert subspace spanned by the projector. Within this framework, the elastic continuum (excited or scattering states) can be recovered.

The crucial point in this approach is to replace the true total potential *V* with the projected potential:

(28) $$V_{\beta }=\sum_{\delta \eta \mu \tau }|g_{\delta }\rangle S_{\delta \eta }^{-1}\langle g_{\eta }\left|V\right|g_{\mu }\rangle S_{\mu \tau }^{-1}\langle g_{\tau }|;\;S_{\delta \eta }=\langle g_{\delta }|g_{\eta }\rangle \;,$$

which results in the truncation of the long-range part of the HF potential. This procedure is based on the idea that it is sufficient to have a projected potential $V_{\alpha }$ that, applied to a plane wave, correctly reproduces the effect of the long range part of the true potential at least in a part of the asymptotic region, where $V_{\alpha }\left(r\right)≃V_{\mathrm{tot}}\left(r\right)$ (when *r* is large and far from the scattering center). This fact guarantees that the scattering wavefunction both inside the molecular volume, which is the important region for calculating the elastic scattering matrix elements, and outside the scattering volume, which determines the normalisation condition, has the correct form. The solution of Equation (26), or equivalently of Equation (27), of course delivers the eigenvalues of the projected Hamiltonian $H_{0}+\pi V\pi$ rather than those of the complete Hamiltonian $H_{0}+V$. However, we notice that the eigenvalues of the projected and complete Hamiltonian coincide, provided that the vectors Vψ and ψ belong to the projected functional subspace.

We used our numerical relativistic approach based on the HF approximation of the wavefunction, along with the Fermi golden rule, to assess the total elastic cross section of electrons moving within liquid water. In particular, due to computational constraints in terms of prohibitive scaling with the system size, we used a cluster of six water molecules to mimic liquid water and account for multiple scattering from the surrounding environment in liquid phase. It is worth to mention here that Hartweg et al. [113] have recently shown experimentally that the photoelectron angular distributions for the valence orbitals of neutral water clusters converges for a size equal or larger than 5–6 molecules, which may indicate that a cluster of such size might already be a good representation of the liquid environment.

These six-molecule cluster has been extracted by the configuration previously obtained by optimising a cell containing 32 water molecules at the experimental density in room conditions. Wavefunctions and potentials were projected in a set of aug-cc-pVTZ Gaussian base functions optimised for both hydrogen and oxygen atoms [114] and centered into the nuclei. Mono- and bi-electronic molecular integrals of the bare Coulomb and exchange interaction are computed at each self-consistent field cycle [46]. Once the problem to find the scattering stationary states of the projected Dirac Hamiltonian $\hat{h}=H_{0}+V_{\alpha }$ at the energy E is solved,

(29) $$\hat{h}|\psi_{k}^{+}\left(E\right)\rangle =E|\psi_{k}^{+}\left(E\right)\rangle \;,$$

the differential cross section for unit solid angle is then obtained as follows:

(30) $$\frac{d\sigma_{\mathrm{el}}^{e-c}}{d\Omega }=\frac{m^{2}}{4\pi^{2}}|\langle \Phi_{k\hat{n}}|T^{+}\left(E\right)|\Phi_{k}{\rangle |}^{2}=\frac{m^{2}}{4\pi^{2}}|\langle \Phi_{k\hat{n}}\left|V\right|\psi_{k}^{+}{\rangle |}^{2}\;,$$

where *m* is the electron mass, $\Phi_{k\hat{n}}$ is the incoming plane-wave impinging on the water cluster with momentum *k* in the direction $\hat{n}$, $\Phi_{k}$ is the outgoing free plane wave elastically scattered in the direction k within the solid angle Ω and (Ω+dΩ), $T^{+}\left(E\right)$ is the on-shell *T*-matrix, *V* is the molecular relativistic potential obtained via the self-consistent solution of the Dirac equation. The scattering wavefunction $\psi_{k}^{+}\left(E\right)$ is characterised by the so-called outgoing (+) wave boundary conditions, which means that the eigensolutions of the Dirac equation asymptotically describe a plane wave plus outgoing spherical waves [46,115].

For swift electrons, one may also adopt the first Born approximation (T=V). Furthermore, since *V* is the approximate representation of the long range Coulomb potential projected on a finite functional space, one can replace $\psi_{k}^{+}$ with $\Phi_{k}$ outside the scattering volume where the potential dies off [44,45,116].

**Figure 6:**
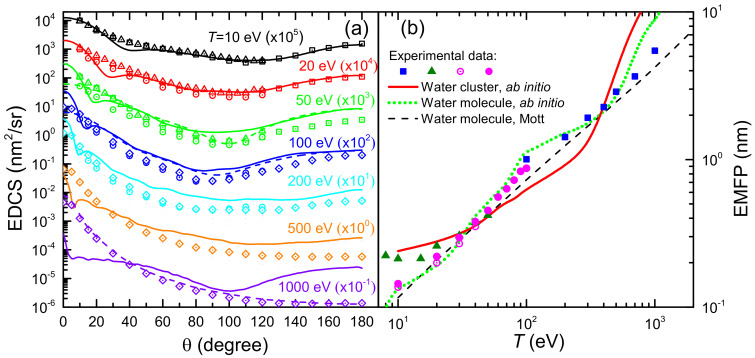
(**a**) Elastic singly differential cross sections (EDCS) of electrons of energy *T* scattered by water molecules and a water cluster. Solid lines correspond to *ab initio* calculations for a cluster of six water molecules [43], while dashed lines are Mott theory calculations for a single water molecule. Symbols are experimental data for water vapour [117] (squares), [118] (circles), [119] (triangles) and [120] (diamonds). (**b**) Elastic free path (EMFP) for electrons in water, as a function of electron energy *T*. *Ab initio* calculations are shown by a red solid line for a cluster of six water molecules [43], by a green dotted line for one water molecule, and by a black dashed line for Mott theory calculations for a water molecule. Symbols represent experimental data for water vapour: [120] (squares), [117] (triangles), [24] (empty circles) and [27] (full circles).

Figure 6a shows the EDCS of electrons incident in liquid water, $d\sigma_{\mathrm{el}}^{e-e}/d\Omega$, for several impinging electron energies (T= 10–1000 eV), i.e., the angular distributions of the electrons emitted at a given angle θ, integrated over the emitted energy, due to elastic collisions. Solid lines correspond to calculations based on the *ab initio* approach for a cluster of six water molecules [43], while dashed lines depict the EDCS calculated for a single water molecule by means of the Mott theory (the latter given for the selected energies 50, 100 and 1000 eV, to avoid the figure to be too crammed). Symbols are experimental data for water vapour (squares [117], circles [118], triangles [119] and diamond [120]). In general, it can be seen how, for this energy range, the Mott theory for the single water molecule gives results in very good agreement with water vapour experiments, as it is to be expected. However, the Dirac–Hartree–Fock calculations for the cluster of six water molecules give, for most of the energies, angular distributions which are significantly different from the single molecule. Clear examples are the energies of 50 eV, where the cluster presents some structure in the EDCS around 20${}^{\circ }$ and a flatter profile around 90${}^{\circ }$, and 1000 eV, at which the EDCS is in general much more isotropic than for the water molecule. It should be noted that experimental techniques actually do not discriminate the elastic cross section from the rotationally inelastic one [24]. Rotational excitations are not accounted for in our calculations, although their contribution are expected to only have an effect at very small angles and, moreover, to have a small impact in the case of liquid water. At low angles, the water molecule polarisability (which is naturally considered in the calculations) may also have a large effect on the elastic scattering in the gas phase [24].

After integration of the EDCS over the solid ngle dΩ around the scattering angle θ of the emitted electron, the total elastic cross section (TECS), depending on the electron energy *T*, is given by:

(31) $$\sigma_{\mathrm{el}}^{e-c}\left(T\right)=\int d\Omega \frac{d\sigma_{\mathrm{el}}^{e-c}}{d\Omega }\;.$$

Notice that from the knowledge of the total elastic cross section one can calculate the macroscopic elastic mean free path (EMFP) for an elastic collision as $\lambda_{\mathrm{el}}=\frac{1}{N\sigma_{\mathrm{el}}^{e-c}}$, where N is the molecular density of the target. In Figure 6b we show the EMFP of electrons in liquid water as a function on their initial kinetic energy *T* in the range 10 eV to 20 keV. The solid red line corresponds to the results obtained from the *ab initio* model for a cluster of six water molecules [43], whereas the green dotted line has been obtained, with the same methodology, for a single water molecule. In addition, it is shown a comparison with experimental data for water vapour from Katase et al. [120] (squares) and Cho et al. [117] (triangles), and with the recommendations by Itikawa and Mason [24] (empty circles) and Song et al. [27] (full circles), as well as a comparison with the Mott theory for the water molecule (black dashed line). All EMFP (calculated and experimental) for the water molecule have been obtained from the molecular cross section but using liquid water molecular density N. Clearly, the Mott theory can excellently reproduce the experimental data on the gas phase (scaled to liquid density) in the entire energy range, at least down to 10 eV. The Dirac–Hartree–Fock calculation for the single water molecule corresponds quite well with the Mott calculation and the scaled vapour data, which validates the approach. However, the *ab initio* calculation for the cluster of six water molecules, as a proxy for the liquid medium, shows clear deviations from the molecular behaviour, particularly in some energy ranges such as below 30 eV or in the range 100–300 eV, which might show the signature of the effect of the liquid environment on the EMFP. It should be noted that, even though the recommended data by Itikawa and Mason [24] and its later revision by Song et al. [27] constitute, up to date, the most complete reference data with which to compare the calculated EMFP in water, some discrepancies with these cross sections have been identifie in a recent simulation study [121], so further research on the elastic (and inelastic) cross sections in water needed.

#### 2.4.3. Inelastic cross Sections of Electrons in Liquid Water

The inelastic cross sections of electrons in liquid water can be obtained from the dielectric formalism (as it was done for carbon ions in Section 2.2 and Section 2.3). However, several factors need to be taken into account [42]. First of all, the free electron travelling in the conduction band of the material is indistinguishable from the valence bound electrons. This fact, together with electron exchange, have to be considered in the calculations, particularly for energies lower than ∼500 eV, where these effects are more noticeable. Second, for electrons with energy <100 eV the first Born approximation, on which the dielectric formalism is based, is not valid any more, and corrections must be in place to increase its accuracy. Finally, very low energy (<30 eV) electrons are not able to ionise all the valence shells. This fact needs to be considered in order to estimate the mean binding energy of the outer-shell electrons, which determines the ionisation and excitation probabilities.

Starting from Equation (13) for ions, the expression for the direct scattering inelastic doubly differential cross section (IDDCS) for electrons, within the FBA, is obtained by just using the electron mass (i.e., taking M=m) and having into account that ${\left[Z-\rho_{q}\left(k\right)\right]}^{2}=1$, as the projectile is a point charge. However, as explained in more details in Ref. [42], an extra exchange term needs to be added for electron projectiles, so IDDCS = IDDCS${}_{\mathrm{FBA}}+$ IDDCS${}_{\mathrm{xc}}$.

Both for electronic excitations and ionisations, Ochkur developed convenient approximations for the exchange factors which retain the FBA component for the direct-scattering amplitude, based on first order Born–Oppenheimer perturbation theory [122,123,124,125]. We have implemented these Born–Ochkur exchange factors for excitations and ionisations [42].

However, the direct FBA expression needs to be corrected for low energy electrons, which was done in Ref. [42] using a simple Coulomb-field approximation [126], so IDDCS = IDDCS${}_{\mathrm{corr}}+$ IDDCS${}_{\mathrm{xc}}$. In practice, the Coulomb-field correction consists on replacing, for the FBA cross section, the electron energy *T* by an energy $T^{\prime }=T+2B_{\alpha }$, where $B_{\alpha }$ is the binding energy of the target electron involved in the excitation [126]. This change takes into account the potential energy gained by the incident electron in the field of the target molecule. In the context of our approximate model for ionisation of liquid water, $B_{\alpha }=B=13.7$ eV for the ionisation of the outer-shell electrons, while $B_{\alpha }$ corresponds to the oxygen K-shell binding energy for the inner-shell ionisation. For the excitation of the outer-shell electrons, the binding energy is approximated as the threshold for electronic excitations, $B_{\alpha }=E_{\mathrm{th}}=7$ eV.

Finally, as anticipated above, very low energy electrons (<30 eV) cannot ionise all the outer-shells of the target. As a consequence, at low energies, the mean binding energy for the valence shells becomes energy dependent, B=B(T). In Ref. [42], B(T) was estimated for several biological targets. For liquid water, it evolves from 10.79 eV (first binding energy) to 13.71 eV (high-energy limit) following a logistic function in the energy range 0–32.3 eV. This correction has a noticeable impact both in the ionisation and (much more remarkably) excitation cross sections below ∼30 eV.

Having into account all these considerations, and integrating the IDDCS over momentum transfer, the energy spectrum of secondary electrons (ionis-SDCS) for electron impact becomes [42]:

(32) dσionise−e(T,W)dW=dσionise−e(T,W)dWcorr+dσionise−e(T,W)dWxc=me2πℏ2N1T+2B(T)∫k−,outk+,outdkkIm−1ϵ(k,W+B(T))outer+∑j1T+2Bj∫k−,jk+,jdkkIm−1ϵ(k,W+Bj)j+1T∫k−,outk+,outdkkFxcionis(T,k,W)Im−1ϵ(k,W+B(T))outer+1T∑j∫k−,jk+,jdkkFxcionis(T,k,W)Im−1ϵ(k,W+Bj)j,

where the first term (’corr’) corresponds to the Born-corrected direct ionis-SDCS, whereas the second term (’xc’) accounts for the exchange ionis-SDCS; the superscript ’e-e’ stands for electron-electron interaction. The Born–Ochkur exchange factor for ionisation is given by $F_{\mathrm{xc}}^{\mathrm{ionis}}\left(T,k,W\right)=-\frac{k^{2}/2m}{T-W}+{\left(\frac{k^{2}/2m}{T-W}\right)}^{2}$. The integration limits in momentum transfer for the Born-corrected terms are [127]:

(33) $$\hbar k_{\pm ,\alpha }=\sqrt{2m(T+2B_{\alpha })}\pm \sqrt{2m\left[\left(T+2B_{\alpha }\right)-\left(W+B_{\alpha }\right)\right]}\;,$$

while for the exchange terms are:

(34) $$\hbar k_{\pm ,\alpha }=\sqrt{2mT}\pm \sqrt{2m(T-E)}=\sqrt{2mT}\pm \sqrt{2m(T-W-B_{\alpha })}\;,$$

with $E=W+B_{\alpha }$, where α=outer/j for the outer/inner-shells.

The integration over energy of the previous expression gives the ionisation total cross section (ionis-TCS) [42]:

(35) σionise−e(T)=σionise−e(T)corr+σionise−e(T)xc=me2πℏ2N1T+2B(T)∫W−,outW+,outdW∫k−,outk+,outdkkIm−1ϵ(k,W+B(T))out+∑j1T+2Bj∫W−,jW+,jdW∫k−,jk+,jdkkIm−1ϵ(k,W+Bj)j+1T∫W−,outW+,outdW∫k−,outk+,outdkkFxcionis(T,k,W)Im−1ϵ(k,W+B(T))out+1T∑j∫W−,jW+,jdW∫k−,jk+,jdkkFxcionis(T,k,W)Im−1ϵ(k,W+Bj)j.

The limits in the integral over the kinetic energy of the emitted electron are $W_{-,\alpha }=0$, which represents the ionisation threshold, either for outer ($B_{\alpha }=B\left(T\right)$) or inner shells ($B_{\alpha }=B_{j}$), and $W_{+,\alpha }=\left(T-B_{\alpha }\right)/2$ limits the amount of energy that the primary electron can lose, originating from the electron indistinguishability: since now both primary and secondary electrons are indistinguishable particles moving in the conduction band, the primary particle cannot end up with less energy than the secondary electron.

On the other hand, electronic excitations can only be produced (within the assumptions of our model) for energy transfers *E* between the excitation threshold $E_{\mathrm{th}}$ and the mean binding energy of the outer shell electrons B(T), as any larger transfer will lead to ionisation. So the excitation total cross section (excit-TCS) will be given by [42]:

(36) σexcite−e(T)=σexcite−e(T)corr+σexcite−e(T)xc=me2πℏ2N1T+2Eth∫E−E+dE∫κ−κ+dkkIm−1ϵ(k,E)outer+1T∫E−E+dE∫k−,outk+,outdkkFxcexcit(T,k)Im−1ϵ(k,E)outer,

where $E_{-}=E_{\mathrm{th}}$ and $E_{+}=\min\left[B(T),T\right]$. As the primary electron moves in the conduction band and the excited target electron is promoted to a lower discrete energy level, indistinguishability does not impose any limit to the amount of energy loss of the former and the maximum energy that it can lose is *T*. The limits in the momentum transfer $\hbar k_{\pm ,\mathrm{out}}$ are given by Equation (34) and $\hbar \kappa_{\pm }=\sqrt{2m(T+2E_{\mathrm{th}})}\pm \sqrt{2m(T+2E_{\mathrm{th}}-E)}$. The Born–Ochkur exchange factor for excitation is given by $F_{\mathrm{xc}}^{\mathrm{excit}}\left(T,k\right)=-\frac{k^{2}/2m}{T}+{\left(\frac{k^{2}/2m}{T}\right)}^{2}$.

**Figure 7:**
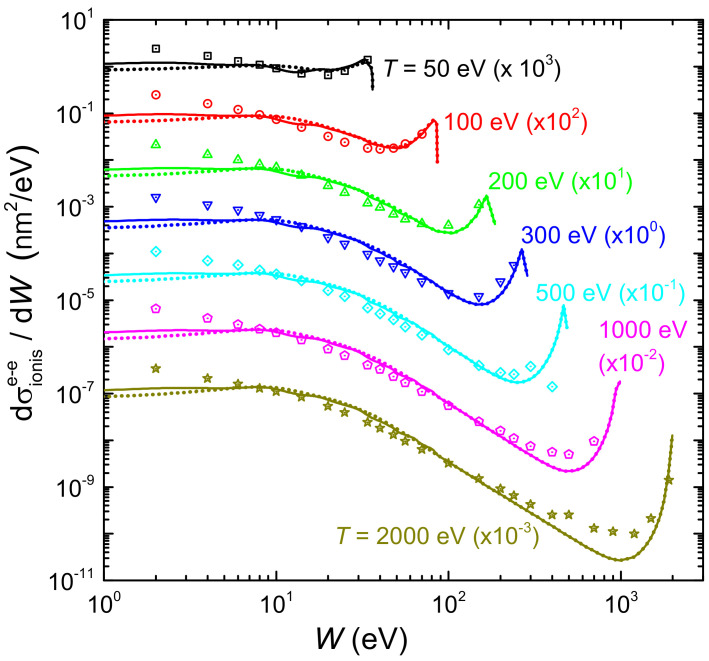
Energy distributions (ionis-SDCS) of electrons generated in water by an initial electron with energy *T*, as a function of their emitted energy *W*. Solid (dotted) lines are calculations obtained with the ELF of liquid water from the LR-TDDFT (MELF-GOS) approach. Symbols are experimental data for water vapour [128].

Figure 7 shows by solid (dotted) lines the ionis-SDCS for electrons of different energies *T* (denoted by labels) impinging on liquid water, as a function of the secondary electron energy *W*, as obtained from the LR-TDDFT (or MELF-GOS) energy loss functions. Symbols correspond to experimental data for water vapour [128]. In general, calculations agree rather well with the entire set of experimental data, particularly for W>10 eV, reproducing the primary peak appearing when W≃T due to primary-secondary electron indistinguishability. Experimental data are systematically larger than calculations for W<10 eV, which could be due to phase effects, or to the difficulty to obtain reliable experimental determinations at this low energy range. Results for the ionis-SDCS based either on LR-TDDFT and MELF-GOS energy loss functions are in general rather similar, with differences being only observed for W<10 eV, where the LR-TDDFT calculations are slightly larger than the MELF-GOS ones.

**Figure 8:**
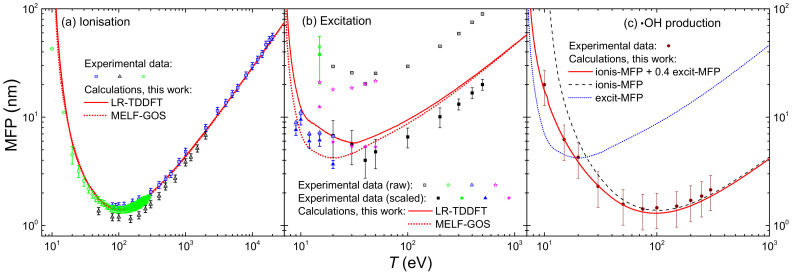
(**a**) Ionisation mean free path (ions-MFP) of electrons in water, as a function of the incident energy *T*. Red solid (dotted) lines are calculations using the LR-TDDFT (MELF-GOS) model for liquid water. Symbols are experimental data for water vapour [129] (blue squares), [128] (black triangles) and [130] (green circles). (**b**) Excitation mean free path (excit-MFP) of electrons in water, as a function of the incident energy *T*. Red solid (dotted) lines are calculations using the LR-TDDFT (MELF-GOS) model for liquid water. Symbols are experimental data for water vapour [131] (black squares), [132] (green circles), [133] (blue triangles) and [134] (magenta starts); empty and full symbols represent raw and scaled experimental data, respectively, as explained in the text. (**c**) Mean free path for the production of ·OH radicals as a function of the incident energy *T*. The red solid line represents a weighting of ionisations and excitations, as explained in the text, based on the MELF-GOS model for liquid water; symbols are experimental data for water vapour [135].

The integration over the secondary electron energy *W* of the ionis-SDCS gives place to the ionisation total cross section (ionis-TCS) or to the related ionisation mean free path (ionis-MFP), whose calculated results are depicted in Figure 8a by a solid (dotted) line as obtained from the LR-TDDFT (MELF-GOS) energy loss function. Again, symbols represent experimental data, only available for water vapour [128,129,130] (scaled with liquid water density to obtain the ionis-MFP), which, despite potential phase-effects, are in excellent agreement with the theoretical calculations. Experimental data are slightly lower than calculations; this is to be expected for the ionis-MFP obtained from the gas-phase cross section as compared to the liquid: the liquid water microscopic cross section is lower than for the vapour due to electronic screening, resulting in a larger ionis-MFP trhough the relation $\lambda_{\mathrm{ionis}}=\frac{1}{N\sigma_{\mathrm{ionis}}^{e-e}}$, where N is the molecular density of the target. For ionisation, the use of the LR-TDDFT or MELF-GOS energy loss function has an almost negligible influence.

The results for the calculated excitation mean free path (excit-MFP) are shown by solid (dotted) lines in Figure 8b as obtained from the LR-TDDFT (MELF-GOS) energy loss function. Contrary to ionisation, the ELF has a strong impact in the excit-MFP, as in this case the low energy transfers are more relevant and, as can be seen in Figure 1, the differences between the ELF models are most significant for energy transfers below 50 eV. The comparison with experimental information for excitation is even more complicated. Not only the available measurements correspond to gas phase water [131,132,133,134], but also each of these datasets is limited to one or a few particular excitation channels and do not refer to the total excitation probability. In Ref. [42], a scaling procedure was suggested to estimate the total excitation cross section from each source of experimental data, based on the most complete available study available for water up to date [25]. Open symbols in Figure 8b correspond to the excit-MFP coming from the original measurements, while full symbols depict the scaled results. Again, the excit-MFP for liquid water is obtained from the microscopic cross section through $\lambda_{\mathrm{excit}}=\frac{1}{N\sigma_{\mathrm{excit}}^{e-e}}$, where the liquid water molecular density is used. As can be seen, scaled experimental data agree rather well with the calculations, which reinforces the theoretical approach and moreover gives support to the scaling procedure applied to the experimental data. The scattered nature of the experimental points makes it difficult to determine the accuracy of these two calculations, but in general it seems that the LR-TDDFT results provide a shape closer to that obtained from the experimental data, being this a fact that one would expect having into account that this ELF is closer to the experimental one [36,63] at low excitation energies than the one provided by the MELF-GOS approach. Experimental excit-MFP are slightly lower than the calculated ones in the entire energy range (even for T>100 eV, where the dielectric formalism is expected to be more reliable), which may indicate some phase-effect differences between the data for a gas and a liquid target, as already discussed above for the ionis-MFP.

Another aspect regarding electronic excitation of water must be stressed. While it is widely assumed that all ionising collisions lead to the dissociation of water molecules in the liquid phase [18,136], not all excitations can fragment them. From the study of Ref. [25], it can be estimated that the excitation channels that contribute to water molecule dissociation constitute around 40% of the total. The dissociation of water molecules leads to a great extent to the production of ·OH radicals. The cross section for ·OH production by electron impact in water molecules was experimentally measured in a wide energy range by Harb et al. [135], whose results for the MFP are presented by symbols in Figure 8c. The MELF-GOS-calculated ionisation and excitation MFP for liquid water are represented in the figure by dashed and dotted lines, respectively. As expected, the ionis-MFP resembles very much the ·OH production MFP for T>30–40 eV, while they depart for lower energies, due to the excitation contribution to water fragmentation. The solid line in the figure represents the MFP calculated from the sum of the theoretical ionisation cross section and 40% of the excitation cross section. Remarkably, this line matches very well the experimental ·OH production MFP, which confirms that ∼40% of the electronic excitations leading to severe effects is a correct estimation. This information will be later used for evaluation of DNA damage by Monte Carlo simulations in the next section. It should be stressed at this point that, even though according to the recommended ionisation data for water molecules [24] not every ionisation event leads to fragmentation, it is customary to assume so in the case of liquid water [18,136]. In any case, the large error bars from the data by Harb et al. [135] prevent us from further considerations on how many ionisations lead to ·OH production, but allow us to support our assumptions regarding dissociative excitations.

### 2.5. Monte Carlo Simulation of Secondary Electron Transport around the Carbon Ion Path

When the carbon ions move through the liquid water target, they generate secondary electrons that deposit energy around the carbon ion path, which results in a carbon ion track structure. We have simulated the transport of these secondary electrons, interacting with the target electrons, with the event-by-event MC code SEED (Secondary Electron Energy Deposition) [43,137,138]. Apart from the elastic and inelastic cross sections explained in previous sections, SEED also implements the electron-phonon interaction and electron-polaron trapping, accounted for by the Fröhlich [139,140] and the Ganachaud and Mokrani [141] models, respectively. These interactions become increasingly more relevant at very low electron energies, and the parameters for the models have been set so MC simulations reproduce the experimentally determined secondary electron yields from liquid water [142,143,144,145,146]. The Ganachaud-Mokrani inverse mean free path for an electron with energy *W* is given by $\lambda_{\mathrm{trap}}^{-1}=C_{\mathrm{trap}}\exp\left(-\gamma_{\mathrm{trap}}W\right)$, where $C_{\mathrm{trap}}=0.1{\;\mathrm{nm}}^{-1}$ and $\gamma_{\mathrm{trap}}=0.1{\;\mathrm{eV}}^{-1}$, while the phonon energy entering the Frölich theory is $W_{\mathrm{ph}}=0.1$ eV. Additionally, very low energy electrons can also damage biomolecules by means of dissociative electron attachment (DEA). In order to account for DEA, its cross section for the water molecule has been taken from recommendations from experimental data [24].

Finally, simulations were typically performed having into account a large amount of electron trajectories, in order to minimise statistical noise, particularly for the damage cluster simulations (to be discussed in the next section). For each carbon kinetic energy *T*, 1200 ion paths of 50 nm length were simulated, with different random seeds at each ion shot to determine the ionisation sites and secondary electron energy and ejection angle. The ion path length was chosen so that virtually all the secondary electrons generated along the carbon ion track can reach the sensitive volume (having dimensions of a DNA-like target), while keeping simulation times within reasonable limits. To achieve an acceptable trade-off between computational cost and low signal-to-noise ratio, 1000 electrons were assumed to be generated initially along the path at each collision between the carbon ion and the water target (this number bearing a purely statistical, and not physical, interpretation). In average, carbon ions undergo 30 (1 GeV) to 1000 (0.2 MeV/u) collisions; thus, each ion shot produces on average $10^{5}$–$10^{6}$ electrons. These electrons produce an average number of 100 further electrons each one, due to further ionisations along their paths. Current simulations are equivalent then to assess 4 to 100 billion electron trajectories per ion energy.

## 3. Results and Discussion

Once the energy and angular distributions of the secondary electrons produced by carbon ion impact on liquid water have been reliably obtained (Section 2.3), together with the relevant elastic (Section 2.4.1 and Section 2.4.2) and inelastic (Section 2.4.3) scattering cross sections for electron transport (particularly relevant for low energy electrons), it is possible to perform detailed simulations of the carbon-ion track-structures (Section 2.5). There are two relevant aspects connected to this point, namely: (i) the radial dose arising from energy deposition around the ion’s path and (ii) the clustering of damaging events being produced in nanometric volumes (mimicking DNA targets) located at different distances from the ion’s path. While the former has been long used as a key input for semiempirical radiobiological models such as the Local Effect Model (LEM) [147], the latter provides much more detailed information, which has proved to be fundamental for the theoretical evaluation of RBE within the MultiScale Approach (MSA) for radiation biodamage induced by ions [5,19,20]. In Section 3.1, the radial doses around carbon ions in liquid water are analysed, while the clustering of damaging events on the nanoscale is assessed in Section 3.2.

### 3.1. Simulation of the Radial Dose in Liquid Water around the Carbon Ion Path

At macroscopic level the most important quantity in radiation therapy is the *absorbed dose*, which is the mean energy deposited in a mass element of tissue by the ionising radiation. The radial dose is an approximation to a microscopic equivalent of the dose, in which the space around the ion path is divided in concentric cylindrical shells of differential width (in the current simulations, dr=1 Å), and the amount of energy deposited in each volume element by secondary electrons is scored and divided by the mass of such element. In this section, we use a detailed Monte Carlo simulation [137,138] to evaluate the influence of different physical events on the radial dose, namely, the effect of different descriptions of the elastic and inelastic cross sections, or the consideration of the carbon ion charge states.

**Figure 9:**
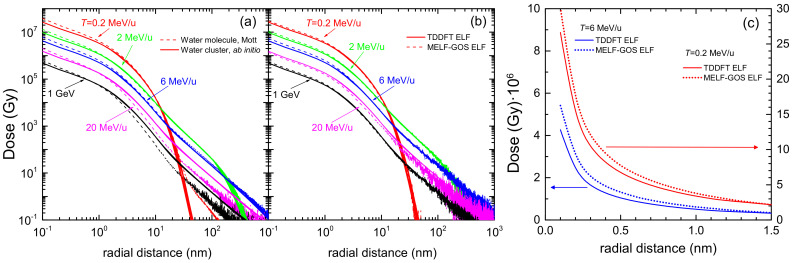
Dose deposited by a carbon ion in liquid water, as a function of the distance from the ion path, for several ion energies *T*. (**a**) Effect of the elastic scattering models when using Mott’s theory for a single water molecule (dashed lines) and *ab initio* calculations for a cluster of six water molecules (solid lines). (**b**) Effect of the inelastic scattering models. (**c**) Detail for the different inelastic models at short radial distances. Solid (dotted) curves correspond to simulations using input data from the LR-TDDFT (MELF-GOS) energy loss function. See the text for further details.

Figure 9 shows the results of radial dose simulations in liquid water around carbon ions of several energies in the range from 0.2 MeV/u to 1 GeV. While initial energies of hundreds of MeV/u are typical in clinical carbon ion beams (so they present ranges of the order of tens of centimetres in tissue), the ions progressively lose their energy while traversing the body, going down to energies of hundreds of keV/u and several MeV/u around the Bragg peak region [148]. On the one hand, 0.2 MeV/u corresponds to a particularly relevant energy of carbon ions around the Bragg peak region, where their biological effects are most severe, as the maximum stopping power occurs at this energy. On the other hand, 1 GeV corresponds to a situation closer to the plateau region of the depth-dose curve. Such energetic carbon ions can also be found in cosmic radiation [149], which represents an important handicap for manned space missions [3].

The influence of the electron elastic scattering cross section model is analysed in Figure 9a, where dashed lines depict simulations performed using the Mott cross section for a single water molecule, whereas solid lines show results from the use of the cross section calculated *ab initio* for a molecular cluster. In both cases, the inelastic cross sections obtained from the LR-TDDFT ELF are used. As can be clearly seen, the choice of the elastic scattering model has a large influence on the radial doses, with sizeable differences at very low radial distance (below 2 nm), but also with large differences at large distances as the ion energy increases.

The influence of the electron inelastic scattering cross section model is studied in Figure 9b. Here, the elastic cross section for the cluster of six water molecules is used in all cases, and results using the inelastic cross sections coming from the LR-TDDFT ELF (solid lines) and from the MELF-GOS ELF (dotted lines) are presented. Although here the differences are not so easily appreciated in the double logarithmic scale, they can be better seen in linear scale in Figure 9c, where the cases of 0.2 and 6 MeV/u carbon ions are shown. Even though the general shape of the curves, their extension in space and their low-dose tail are very similar, still considerable differences in the doses at very short radial distances (<1 nm) can be observed. These can be, locally, as large as $10^{6}$ Gy or up to 25% in relative terms.

**Figure 10:**
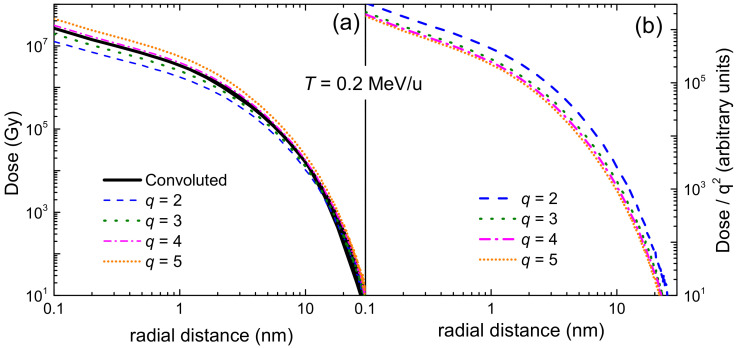
(**a**) Dose deposited by 0.2 MeV/u carbon ions in liquid water, as a function of the radial distance from the ion path, for different charge states *q* of the ion, obtained with the LR-TDDFT model of the ELF. (**b**) The same quantity, divided by the square of the ion charge, in arbitrary units.

Another aspect which can importantly affect the energy deposition patterns around ion paths is the charge state distribution of the ions. This is especially relevant for carbon, as it can have many different charge states at the energies characteristic of the Bragg peak region. Figure 10a depicts the radial doses for 0.2 MeV/u carbon ions, simulated assuming that the carbon ion has a definite charge state from q=2 to q=5 (for the possible charge states of carbon at this energy, see Figure 2), or calculated as a convolution of all the charge states weighted by their corresponding charge fractions. Even though their shape is always similar, the absolute scale of the dose is rather different (except for large radial distances), due to the increasing number of secondary electrons produced by the more charged ions. Some models and simulations in the literature approximately account for this fact by scaling the inelastic cross sections by the square of an energy-dependent effective charge $q_{\mathrm{eff}}^{2}\left(T\right)$. In Figure 10b, the simulated radial doses have been divided by the square of each charge state, in order to assess if the differences are exclusively due to the charge-square scaling in the ejection of secondary electrons. Even though some of the curves converge by this normalisation, not all of them do. This fact remarks that, for carbon ion track-structure simulations, it is important to take into account the detailed charge state distribution of the ion.

**Figure 11:**
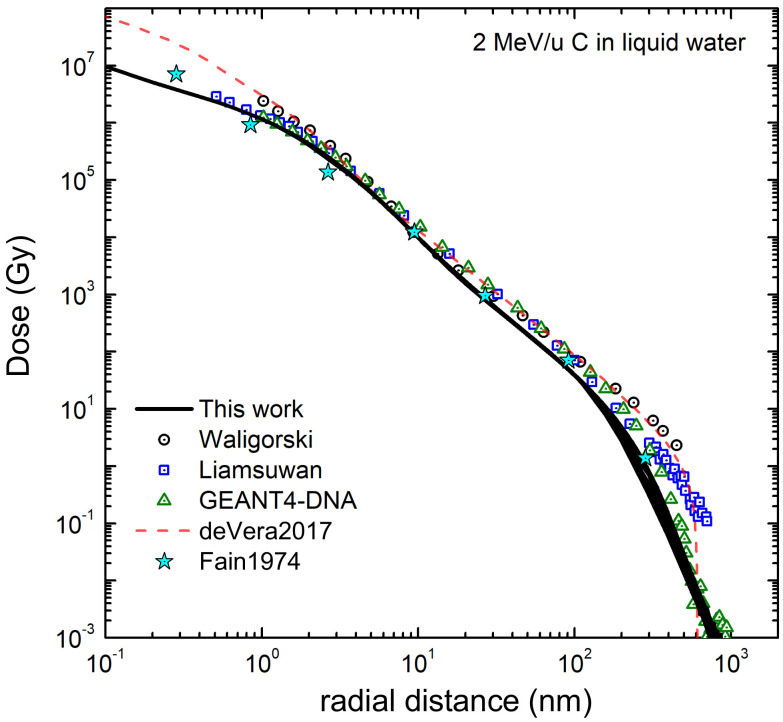
Dose deposited by 2 MeV/u carbon ions in liquid water, as a function of the radial distance from the ion path. Our simulation results are shown by a solid black line, corresponding to the *ab initio* models for liquid water, both for elastic and inelastic collisions. Comparison with other simulations are presented: [152] (circles), [153] (squares) and [154] (triangles). The result of an analytical model for liquid water is also presented (dotted line) [150]. Stars are calculations from Ref. [151] (as they appear in Ref. [152]).

Finally, all three previously described features are taken into account together in Figure 11, where the simulated radial dose for 2 MeV/u carbon ions in liquid water can be compared with several other calculated [150,151] and simulated results [152,153,154]. Our simulations (which are performed with the cross sections derived from *ab initio* calculations, both for elastic and inelastic scattering) are rather consistent with other recent simulations, such as those by Liamsuwan et al. [153] or from Geant4-DNA [154], except for some differences at very large radial distances. However, large differences can be observed at short distances with respect to the classical simulations by Waligorski et al. [152] or from the analytical calculations from de Vera et al. [150], which remarks the need to count with the most accurate cross sections for conducting reliable simulations at the nanometre scale.

### 3.2. Simulation of Clustered Damage on the DNA Strand Scales

Apart from the radial doses, MC codes give access to much more detailed information relevant for determining radiation biodamage. Particularly, the clustering of damaging events in volumes of the dimensions of a few DNA strand twists is in the core of the estimation of RBE by means of theoretical models, such as the MultiScale Approach [5,19,20] or experimentally by means of nanodosimetry [21,22,23].

**Figure 12:**
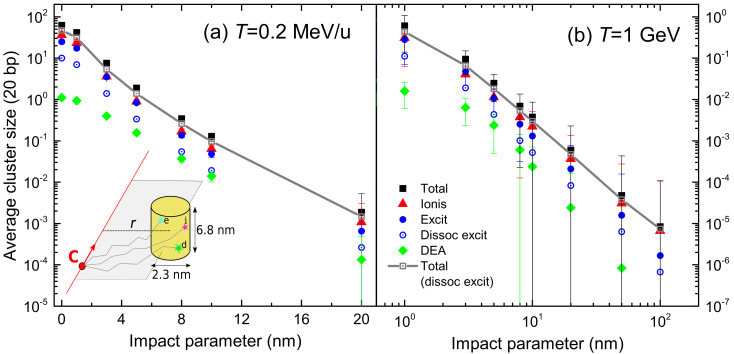
Average cluster size in a sensitive volume of liquid water having the dimension of two DNA turns, as a function of the impact parameter *r* from the ion path. Results for two kinetic energies are depicted: (**a**) *T* = 0.2 MeV/u and (**b**) *T* = 1 GeV. Symbols correspond to clusters produced by a single type of event: ionisations (red triangles), excitations (blue circles), dissociative excitations (blue hollow circles), dissociative electron attachments (DEA) (green diamond). The total average cluster size including all the events is shown by black squares, while that only including the damaging events is depicted by gray squares. The letters in the inset of panel (a) representing the sensitive cylindrical volume refer to excitation (e), ionisation (i) and DEA (d).

The SEED code [137,138], being an event-by-event MC program fed with reliable elastic and inelastic cross sections for liquid water, allows determining the distributions of damaging events being produced in nanovolumes similar to the dimensions of two convolutions of the DNA molecules, namely, a strand 20 base-pair long. Such a strand is modelled as a cylinder of 2.3 nm diameter and 6.8 nm height, whose centre is placed at different distances (or impact parameters) from the ion path, with the symmetry axis along the perpendicular direction, as schematically depicted in the inset of Figure 12a. DNA molecules of such dimensions are frequently considered as relevant sensitive biological targets in radiobiological studies.

By MC simulation, the average size of a cluster of damaging events can be obtained, produced by the different direct damaging interactions, namely, ionisations, electronic excitations and DEA. For these detailed simulations, the most advanced sets of elastic and inelastic cross sections, based on the *ab initio* models described in the previous sections, are used. The average cluster size due to the different events, for impact parameters ranging from 0 to 100 nm, are represented in Figure 12a,b for the lowest and highest energies studied in this work, 0.2 MeV/u and 1 GeV, respectively. Error bars, which in most cases are smaller than the symbols size, represent the statistical uncertainties obtained from the large number of simulations.

As it can be seen, ionisations (triangles) typically dominate for all impact parameters, with electronic excitations (full circles) being always close. However, it should be kept in mind, as discussed in Section 2.4.3, that only around 40% of the electronic excitations are capable of dissociating water molecules and, thus, of inducing severe damage. Empty circles show the average size of these dissociative excitations clusters, clearly much smaller than ionisation clusters. Finally, diamonds depict the average size of damaging clusters produced exclusively by DEA events which, as can be clearly seen, are almost two orders of magnitude smaller than ionisation clusters. Full and empty squares represent the total average size of the clusters, taking into account all excitations or only dissociative excitations, respectively. In general, we will only consider the latter (i.e., 40% of the excitations) to contribute to the cluster of damaging events. For 0.2 MeV/u carbon ions, the total damage clusters are around two orders of magnitude larger than for 1 GeV carbon ions at the shorter impact parameters; these differences tend to disappear for the larger distances. For example, at 1 nm the average cluster is of 31 total damaging events, while for 1 GeV it is 0.44.

**Figure 13:**
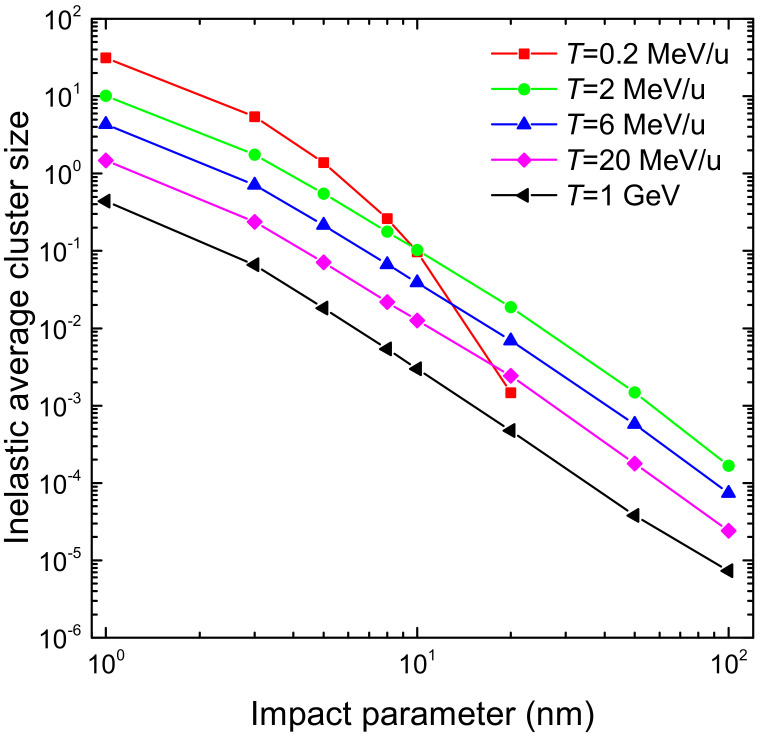
Average cluster size in a sensitive volume of liquid water having the dimension of two DNA turns, as a function of the impact parameter *r* from the ion path, for different values of the carbon ion energy *T*. Ionisations, dissociative excitations and dissociative electron attachments events are taken into account.

The average total sizes of clusters of damaging events (i.e., only considering dissociative excitations as damaging excitation events) are shown in Figure 13 for several energies ranging from 0.2 MeV/u to 1 GeV and for impact parameters from 1 to 100 nm. Clearly, these distributions follow very closely the shape of the radial doses represented in Figure 9. For example, as not very energetic delta electrons are produced for the case of 0.2 MeV/u carbon ions, giving place to a radial dose falling off around 30 nm, the cluster sizes abruptly drops to zero at this distance. For other energies, the decrease is more monotonic, again following the shape of the radial doses shown in Figure 9. For 0.2 MeV/u ions, the average cluster sizes are significantly large (≥10) at ion-target impact parameters below 3 nm, and are always larger than 1 for distances lower than 5 nm. Cluster-size distributions progressively decrease at increasing ion energies, being still larger than 1 at energies below 6 MeV/u at impact parameters closer than 3–4 nm for 2 MeV/u and 2 nm for 6 MeV/u. 1 GeV ions are not capable of inducing clusters of average size larger than 1 for any impact parameter. These behaviours clearly illustrate the different features, in terms of capacity to induce complex damage, of ions with energies characteristic of the Bragg peak region, for the lower ones, or of the Bragg peak plateau, for the larger ones.

In experimental nanodosimetry, frequently the only events which can be straightforwardly measured are ionisations. Apart from the average ionisation cluster size, it is common to find in the literature compilations of cumulative ionisation distributions $F_{n}^{\mathrm{ionis}}$, which measure the probability of inducing in a given sensitive volume a cluster of size equal or larger than *n*. This quantity is relevant, as $F_{2}^{\mathrm{ionis}}$ is correlated to the probability of inducing DNA double strand breaks, or $F_{3}^{\mathrm{ionis}}$ to that of inducing more complex lethal damage.

**Figure 14:**
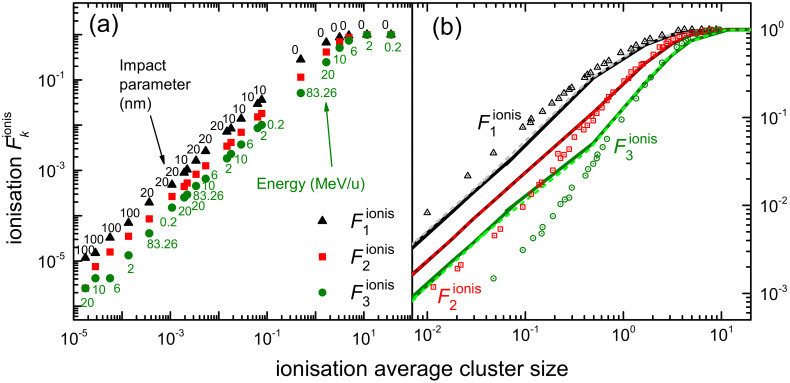
(**a**) Simulated results for the cumulative distributions of ionisation clusters $F_{n}^{\mathrm{ionis}}$ (*n* = 1, 2, or 3), as a function of the average ionisation cluster size. Labels close to each triad of symbols denote the corresponding impact parameter and energy of the ion used in the simulation. (**b**) Experimental data compilation (symbols) for the cumulative distributions of ionisation clusters, as a function of the average ionisation cluster size [23], compared to our simulated results (lines). Dashed lines correspond to a nanometric cylinder similar to the dimensions of a 10-base-pair DNA convolution, while solid lines correspond to a 20-base-pair cylinder.

Remarkably, it is known that the representation of the measured $F_{n}^{\mathrm{ionis}}$ distributions as a function of the average ionisation cluster size yields a universal distribution independent of the size and characteristics of the particular nanodosimeter, which can be used to predict cell inactivation cross sections [22,23]. We check this behaviour by simulation with the results presented in Figure 14a, where we have plotted $F_{n}^{\mathrm{ionis}}$ versus the average ionisation cluster size for a collection of simulations featuring different carbon ion energies and impact parameters. In the figure, different symbols correspond to the different cumulative distributions $F_{n}^{\mathrm{ionis}}$, and numbers next to each triad of symbols inform about the energy and impact parameter used in the simulations. An increase in the ion energy naturally leads to a decrease in the average cluster size, which is accompanied by the decrease of the different $F_{n}^{\mathrm{ionis}}$ distributions. These nanodosimetric quantities also decrease, for each energy, with the increase of the impact parameter.

The simulated points in panel (a) of Figure 14 reproduce the universal curve observed experimentally, as it can be seen in panel (b), where our results are directly compared with a compilation of experimental data [22,23]. Even though these data comes from different nanodosimeters of diverse composition, size, etc., of course different to our simulation setup, the universality of the curve is confirmed by the rather good agreement between simulations and experiments in a wide range of conditions. The agreement is particularly good in the region where the largest clusters are formed, corresponding to impact parameters lower than or around 10 nm for all simulated energies.

**Figure 15:**
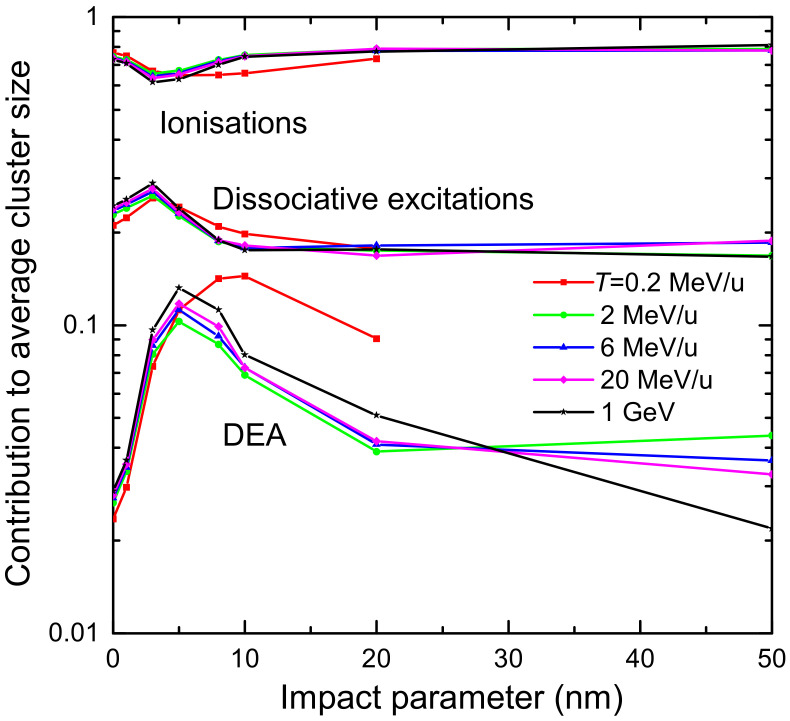
Fractional contribution of different physical mechanisms (ionisations, dissociative excitations, DEA) to the average cluster size in a cylinder with a dimension of two DNA turns for several carbon-ion kinetic energies and impact parameters.

Finally, having into account that SEED simulations reproduce fairly well the experimental ionisation cluster size distributions, and that the code can account for the damage produced also by dissociative electronic excitations and dissociative electron attachment (DEA), it would be interesting to assess the relative contribution of each physical mechanism to the induction by carbon ions of direct radiation damage to liquid water sensitive volumes (having the characteristics of DNA-like targets). The relative contribution (ionisation, dissociative excitations, DEA) to the average size of the damage clusters is plotted in Figure 15 for different ion energies, as a function of the impact parameter. As can be seen, the picture is rather similar for all energies, with only the case of 0.2 MeV/u carbon ions slightly departing from the rest. For impact parameters ≥20 nm, the relative contributions are rather constant, with ionisations providing around 80% of the cluster size, followed by dissociative excitations that furnish around 15–17%, and with DEA only contributing around 5% or less. The percentages of dissociative excitations and DEA grow for short impact parameters (≤10 nm), presenting maxima of around 30% at 3 nm and 10–15% at 5 nm, respectively, at the expense of ionisations, which decrease to 60–65% at 3–5 nm. In light of these results we can safely state that ionisation events make up for the largest contribution to the clustered direct damage induced by carbon ions in liquid water DNA-like targets, which supports the use of ionisation-based nanodosimeters. DEA, typically regarded as a very relevant biodamage mechanism in electron-beam related processes [17], surprisingly plays a minor role in carbon-ion induced clusters of harmful events, according to the present simulations.

## 4. Summary and Conclusions

In this work, the calculation of inelastic and elastic scattering cross sections for carbon ions and their secondary electrons in liquid water, by means of advanced *ab initio* and semiempirical methods has been reported in detail. In particular, inelastic (electronic) cross sections for carbon ions and electrons have been obtained in the framework of the dielectric formalism, exploiting two approaches to describe the electronic excitation spectrum of liquid water: the MELF-GOS method based on the extension of experimental optical data over the entire Bethe surface, and the LR-TDDFT method allowing the first principles calculation of the excitation spectrum. Electron elastic scattering cross sections have been obtained by means of the widely used Mott model and within the Dirac–Hartree–Fock approach, for a single water molecule and for a water molecule cluster, in order to approximate the condensed phase of the target. Simulations of the patterns of energy deposition and clustering of damaging events on the nanoscale have been conducted by means of the SEED (Secondary Electron Energy Deposition) Monte Carlo code, implementing the developed cross sections.

The calculation of the energy loss function (ELF) of liquid water has been explained in Section 2.1. The MELF-GOS method (Section 2.1.1) has been considered a very successful approach during the last years to approximate the excitation spectrum of liquid water, but relies on the availability of optical experimental data. In turn, LR-TDDFT (Section 2.1.2) has matured as a very reliable method to predict such information from first principles. We have shown how LR-TDDFT can almost perfectly reproduce the experimental determinations of the ELF for arbitrary momentum transfers, giving results even more accurate than the MELF-GOS method. However, performing LR-TDDFT calculations for arbitrarily large energy and momentum transfers is prohibitive, and hence the MELF-GOS methodology still is required to extend *ab initio* determinations for energies ≥100 eV and momenta larger than ≥2.5 a.u.

The energy loss of carbon ions is calculated within the dielectric formalism in Section 2.2, having into account the main particularity of this projectile, namely, the large number of charge states (from 0 to 6) that it can present. Energy-dependent charge state fractions have been obtained by means of the CasP semiempirical approximation, which is shown to deliver results consistent with the scarce experimental data. While carbon ions with energies larger than 3 MeV/u (in the range of the clinical ones) travel in liquid water as bare C${}^{6+}$ ions, when they slow down and get energies more typical of the Bragg peak region (several hundreds keV/u), the species from C${}^{2+}$ to C${}^{6+}$ coexist. This fact has been taken into account for obtaining the “macroscopic” cross sections of carbon ions in liquid water, namely, the inverse inelastic mean free path, the stopping power and the energy-loss straggling. Our calculations both from the MELF-GOS and LR-TDDFT energy loss function provide values that agree well with the most recent experimental determination around the maximum of the stopping power curve.

The energy and angle spectra of secondary electrons produced by carbon ions in liquid water are obtained in Section 2.3 within the dielectric formalism. They are in very good agreement with the also scarce experimental information available, unfortunately only for single water molecules. While the angular distributions are not affected much by the ELF model, the energy distributions present a slightly larger contribution of electrons having energies W<10 eV when the LR-TDDFT ELF is used instead of the MELF-GOS one.

The modelling of the carbon ions track-structure also requires accurate cross sections for (secondary) electrons in liquid water, obtained in Section 2.4. The Mott theory (Section 2.4.1) is applied for the elastic scattering with a single water molecule, while the Dirac–Hartree–Fock approach (Section 2.4.2) is also used for a cluster of six water molecules. Differential and integral cross sections for the single water molecule agree very well with the experiments for water vapour (validating both models), with the Dirac–Hartree–Fock method reproducing slightly better the general shape of the integral cross section. However, sizeable differences appear when applying the Dirac–Hartree–Fock method to a water molecule cluster. Differential cross sections become slightly less structured and more isotropic, and the elastic mean free path presents an intense reduction between 100 and 300 eV, and an increase below 30 eV, which seem to arise owing to the phase effects on the elastic cross sections.

The inelastic cross sections for electrons in liquid water are obtained in Section 2.4.3 by adapting the dielectric formalism to the particularities of low energy electron projectiles. Both the MELF-GOS and the LR-TDDFT energy loss functions give results in very good agreement with a compilation of experimental data for electronic excitation and ionisation. While the ionisation cross section is not much dependent on the ELF model considered, the excitation cross section is rather sensitive. The LR-TDDFT ELF helps to increase the excitation mean free path in the energy range 7–100 eV, further approaching the calculated results to the experimental data. For the electronic excitation cross sections, the fraction of them which is capable of inducing water molecule fragmentation has been estimated from the available experimental information [25], giving place to a calculated cross section for ·OH radical production in excellent agreement with experiment.

Track-structure simulations of carbon ions in liquid water in a wide energy range, covering from the low energies characteristic of the ions in the Bragg peak region (0.2 MeV/u, where the maximum energy loss occurs, as well as several MeV/u energies) up to high energies typical from the Bragg curve plateau or of cosmic radiation (e.g., 1 GeV), have been performed by the SEED code (Section 2.5). Apart from elastic and inelastic scattering, SEED also implements dissociative electron attachment (DEA) and other quasi-elastic and trapping events, namely, electron-phonon and electron-polaron interactions.

Radial doses deposited by secondary electrons around the carbon ion paths are simulated (Section 3.1), showing how the patterns of energy deposition concentrate more and more as the energy of the ions approaches 0.2 MeV/u (Bragg peak region). The role of the different elastic and inelastic scattering models on the radial doses has been analysed. While the impact of the inelastic cross sections (coming either from the MELF-GOS or LR-TDDFT ELF) is lower, still differences of up to 25% in the amount of dose at very short radial distances (<1 nm) can be observed. The impact of the elastic scattering model (single water molecule versus water molecule cluster) is more visible, with noticeable changes in the magnitude of the radial doses at all distances. The radial doses have also been simulated for several charge states of the carbon ions. It is found that, in general, the radial dose cannot be simply scaled by the square of the charge of the ion, which advises for an accurate consideration of the charge states instead of resorting to simpler approaches such as energy-dependent effective charges.

The most accurate set of cross sections (coming from the *ab initio* approaches) have been used to assess the clustering of damaging ionisations, dissociative excitations and DEA on nano-cylinders mimicking 20-base-pair DNA strands around the ion paths (Section 3.2). The trends of the average cluster size distribution resembles that of the radial doses, with the higher energy ions (T>6 MeV/u) not being capable of producing clusters of sizes larger than 1 for most of the impact parameters, and with the ions with energies close to the Bragg peak region inducing clusters of tens of events at close enough impact parameters (<10 nm). The universal relation (independent of the energetic ion and nanodosimeter used) between the cumulative distributions of ionising events and the average ionisation cluster size observed experimentally [22,23] has been confirmed by our simulations, which agree with the experimental data fairly well in a wide range of energies and impact parameters.

Finally, even though experimentally ionisations remain the only event to be straightforwardly measured, our simulations allow to assess the relative contribution of the different physical mechanisms to the induction of direct clustered damage by carbon ions. It is found that, indeed, ionisations contribute between 60% and 80% to the average cluster size, almost independently of the ion energy and impact parameter analysed. They are followed by dissociative excitations, which contribute between 15% and 30%. Surprisingly, DEA only have a limited role (around 3% to 15%) in the induction of direct clustered damage by carbon ions. These results provide reassurance on the use of ionisation-based nanodosimeters for the estimation of direct clustered damage produced by carbon ion beams.

## Data Availability

The datasets generated during the current study are available from the corresponding author on reasonable request.

## Abbreviations

The following abbreviations are used in this manuscript:

DEA	Dissociative electron attachment
DFT	Density functional theory
ELF	Energy loss function
EMFP	Elastic mean free path
EDCS	Elastic differential cross section
excit-MFP	Excitation mean free path
excit-TCS	Excitation total cross section
IDDCS	Inelastic doubly differential cross section
IIMFP	Inverse inelastic mean free path
ionis-DDCS	Ionisation doubly differential cross section
ionis-MFP	Ionisation mean free path
ionis-SDCS	Ionisation singly differential cross section
ionis-TCS	Ionisation total cross section
LR-TDDFT	Linear-response time-dependent density functional theory
MC	Monte Carlo
MELF-GOS	Mermin Energy Loss Function – Generalised Oscillator Strengths
MFP	Mean free path
RBE	Relative biological effectiveness
SEED	Secondary Electrons Energy Deposition
